# Transition state-based ST6Gal I inhibitors: Mimicking the phosphodiester linkage with a triazole or carbamate through an enthalpy-entropy compensation

**DOI:** 10.1038/s41598-017-14560-0

**Published:** 2017-10-31

**Authors:** Andrew P. Montgomery, Danielle Skropeta, Haibo Yu

**Affiliations:** 1 School of Chemistry, Faculty of Science, Medicine and Health, University of Wollongong, Wollongong, NSW 2522 Australia; 2 Centre for Medical and Molecular Bioscience, University of Wollongong, Wollongong, NSW 2522 Australia; 3 Illawarra Health and Medical Research Institute, Wollongong, NSW 2522 Australia

## Abstract

Human β-galactoside α-2,6-sialyltransferase I (ST6Gal I) catalyses the synthesis of sialylated glycoconjugates. Overexpression of ST6Gal I is observed in many cancers, where it promotes metastasis through altered cell surface sialylation. A wide range of sialyltransferase inhibitors have been developed, with analogues structurally similar to the transition state exhibiting the highest inhibitory activity. To improve synthetic accessibility and pharmacokinetics of previously reported inhibitors, the replacement of the charged phosphodiester linker with a potential neutral isostere such as a carbamate or a 1,2,3-triazole has been investigated. Extensive molecular dynamics simulations have demonstrated that compounds with the alternate linkers could maintain key interactions with the human ST6Gal I active site, demonstrating the potential of a carbamate or a 1,2,3-triazole as a phosphodiester isostere. Free energy perturbation calculations provided energetic evidence suggesting that the carbamate and 1,2,3-triazole were slightly more favourable than the phosphodiester. Further exploration with free energy component, quasi-harmonic and cluster analysis suggested that there is an enthalpy-entropy compensation accounting for the replacement of the flexible charged phosphodiester with a neutral and rigid isostere. Overall, these simulations provide a strong rationale for the use of a carbamate or 1,2,3-triazole as a phosphodiester isostere in the development of novel inhibitors of human ST6Gal I.

## Introduction

Sialic acid (*N*-acetylneuraminic acid, Neu5Ac) is one of the body’s most important sugars next to glucose^1^. Sialylation, the addition of sialic acid to cell surface molecules via the glycosyltransferase (GT) enzymes known as sialyltransferases (STs), is integral to cell function, governing numerous important biological processes including cell–cell recognition, protein targeting, cell adhesion, and fertilisation^2–4^. However, abnormal sialylation is strongly associated with cancer, with hypersialylation of up to 30–40% seen in many cancers, along with marked upregulation of ST activity. This has been directly correlated with an increased metastatic potential of tumours and poor patient prognosis^5,6^. Upregulation of STs has also been shown to produce cellular resistance in both paclitaxel and cisplatin treatment in human ovarian and colorectal cancer cell lines, along with reducing treatment efficacy^7^. Thus the critical role of STs in the process of tumour metastasis as well as drug resistance demonstrates that inhibitors of STs could be used either alone as anti-metastatic agents, or in combination with existing drugs to enhance their sensitivity against resistant tumours^8^.

In humans, there are 20 different STs each of which share a common sugar nucleotide donor, cytidine 5ʹ-monophosphate Neu5Ac (CMP-Neu5Ac)^2,9^. STs are classified into one of four groups based on the position and identity of the glycosyl acceptor (galactose, *N*-acetylgalactosamine or sialic acid) to which sialic acid is transferred: ST3Gal I-VI, ST6Gal I-II, ST6GalNAc I-VI, and ST8Sia I-VI^2,3^. Each ST subtype controls the synthesis of specific sialylated structures with unique biological roles, therefore it is crucial that selective inhibitors are developed.

Herein we focus on human β-galactoside α-2,6-sialyltransferase I (ST6Gal I), which is one of the two subfamilies of the ST6 family that belongs to the GT29 family (http://www.cazy.org)^10^. ST6Gal I catalyses the transfer of a Neu5Ac residue to the 6ʹ-hydroxyl group of the terminal Gal residues of the disaccharide Galβ[1,4]GalNAc^3,11^. ST6Gal I mediated sialylation can modulate protein conformation, oligomerisation and/or receptor internalisation^12^, depending on the substrate targeted. Overexpression of ST6Gal I is observed in several types of cancers including lung, cervical, ovarian, pancreatic, breast and colon carcinoma^13–16^. It has been recently reported that ST6Gal I promotes a cancer stem cell (CSC) phenotype, with the activity of ST6Gal I critical for CSC behaviours including tumour spheroid growth, chemoresistance and tumour initiating potential^16^. Additionally, overexpression of ST6Gal I has been suggested to promote cancer cell metastasis through altered sialylation patterns affecting the function of β1 integrin^17,18^. ST6Gal I also has an important role in galectin regulation, via the sialylation of their galactose-containing substrates^12,13^. For instance, α-2,6 sialylation of the galectin substrates Fas^19^ and TNFR1^20^ death receptors have been shown to hinder the apoptosis process, thus identifying ST6Gal I overexpression as an inhibitor of cell death pathways. It has also been recently demonstrated that ST6Gal I has a role in promoting tumour cell survival within serum-depleted environments, such as those within the hypovascularised regions of large, solid tumours^21^.

**Figure 1:**
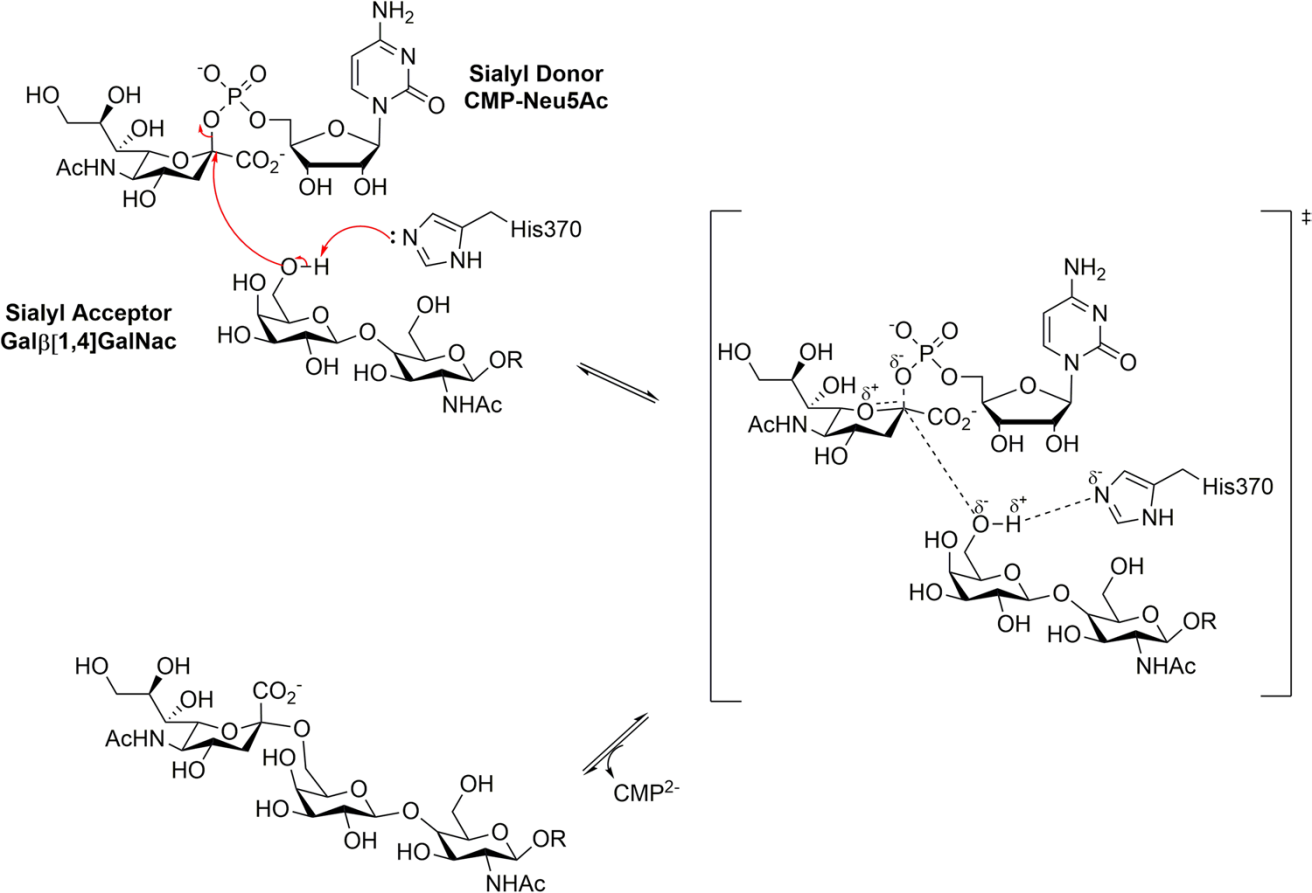
Proposed reaction scheme for the catalytic mechanism of human ST6Gal I. R represents a glycan chain of a glycoconjugate. Replicated from Montgomery, A., Szabo, R., Skropeta, D. & Yu, H. Computational characterisation of the interactions between human ST6Gal I and transition-state analogue inhibitors: insights for inhibitor design. *J. Mol. Recogn* **29**, 210–222, doi:10.1002/jmr.2520 (2016) with permission from Wiley.

The catalytic mechanism of human ST6Gal I (Fig. 1) follows a classical S_N_2-like direct displacement mechanism for inverting GTs^11,22^. It is facilitated by the catalytic base His370, which promotes the nucleophilic attack on the sialyl donor anomeric carbon by deprotonating the galactose 6ʹ-hydroxyl of the acceptor^23,24^. This generates an oxocarbenium-like transition state (TS), followed by CMP acting as a leaving group. The β-configuration at the anomeric carbon of the donor is inverted in the final product^24^. This is supported by a model of the Michaelis complex generated from the glycan binding mode observed in the crystal structure of human ST6Gal I^25^.

**Figure 2:**
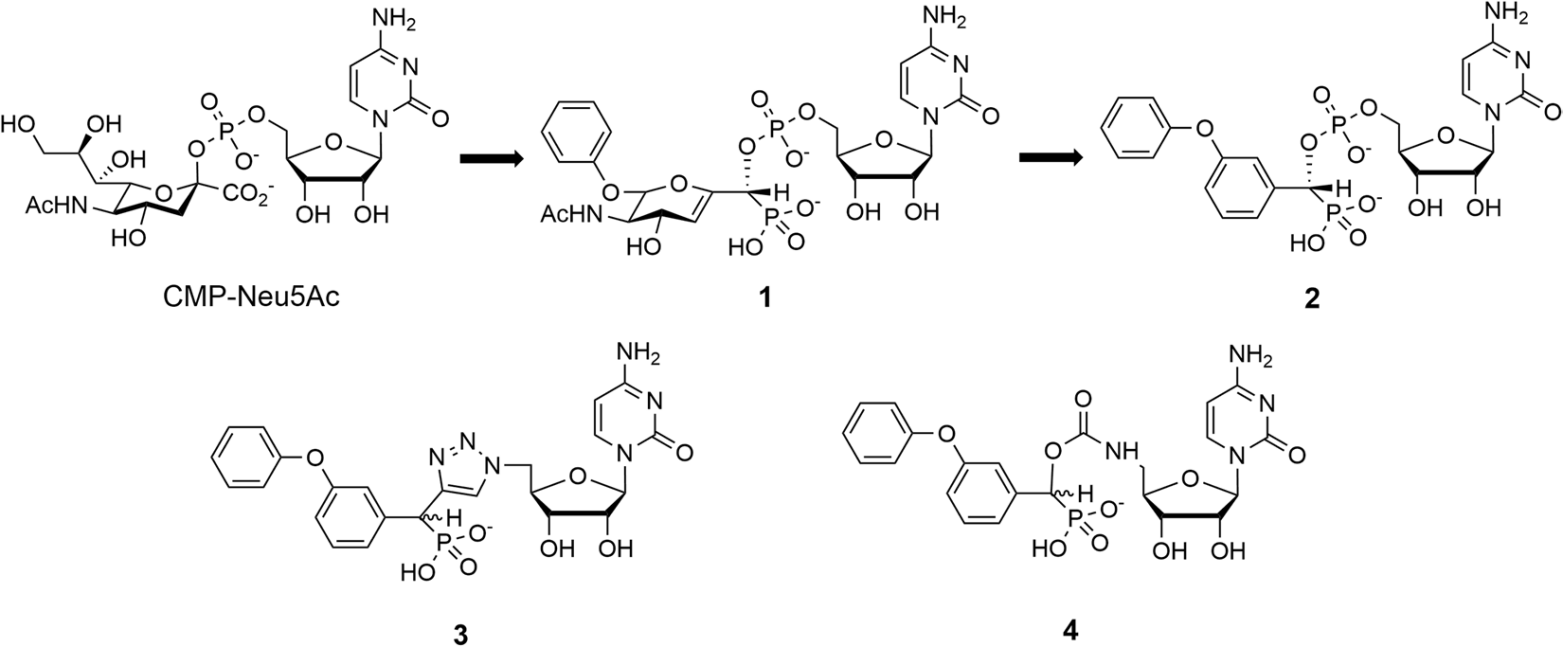
Development of the phosphodiester-linked lead compound (2) and the analogous 1,2,3-triazole- (3) and carbamate- (4) linked derivatives.

The overexpression of ST6Gal I in various cancers has resulted in the development of a diverse range of ST inhibitors^26–28^. Of these, analogues mimicking the proposed oxocarbenium ion-like TS of the donor (Fig. 1) exhibit the highest affinity to STs^29,30^. TS analogue inhibitors incorporating a 2-deoxy-2,3-didehydro-*N*-acetylneuraminic acid (Neu5Ac2en) moiety are amongst the most potent inhibitors. The TS-like characteristics are captured with the C2-C3 double bond mimicking the planar anomeric carbon and an extra carbon between the anomeric carbon and CMP leaving group to mimic the elongated distance in the TS^29,31^. Various strategies have been adopted to increase the potencies of these inhibitors. Derivatives which explore variations of the glycerol side chain of the Neu5Ac2en moiety produced the greatest inhibitory activity. Of these, phenoxy derivative **1** (Fig. 2) is one of the most potent TS analogue inhibitors reported to date (*K*_*i*_ = 29 nM for rat liver α-2,6-ST)^32^. More synthetically assessable derivatives that incorporate an aryl replacement of the Neu5Ac2en moiety have comparable activity to the phenoxy derivative **1** ^29,30,33^. Of these the (*R*)-isomer of the phenoxy derivative **2** (Fig. 2) was a potent inhibitor of rat liver α-2,6-ST (*K*_*i*_ = 70 nM)^29^ and human ST6Gal I (*K*_*i*_ = 19 nM)^34^ and is the lead compound herein. Recently, cyclopentyl^34^ and amide^35^ ST inhibitors have been reported with comparable nanomolar activity to the phenoxy lead derivative (**2**) against human ST6Gal I.

Many of the above inhibitors were designed with limited knowledge of the target ST structure. The recent characterisation of the mammalian ST crystal structures, in particular that of human ST6Gal I^25^ and human ST8Sia III^36^, has facilitated the use of computational modelling to aid in the design of new selective ST inhibitors. In this work, we use computational modelling to investigate the effect of replacing the charged phosphodiester linkage of TS analogue ST inhibitors. This is being investigated, as while contributing to potency^37^ the phosphodiester linkage may potentially contribute to low bioavailability, loss of activity due to cleavage by phosphatases or instability in the ST active site^38,39^. To build upon our previous work^40,41^, molecular dynamics (MD) simulations and binding free energy calculations via the free energy perturbation (FEP) method will be undertaken to investigate the effect of replacing the phosphodiester linker of our lead compound **2**, with either a 1,2,3-triazole (**3**; Fig. 2) or carbamate (**4**; Fig. 2), has on binding to human ST6Gal I. Force field parameters compatible with the CHARMM PARAM36 force field for the ligands will be developed^42^. Through equilibrium MD simulations the conserved interactions as well as potential differences between each compound will be compared. Furthermore, relative binding free energy calculations will be used to study the effect of the linker on binding affinity.

## Methods

### Force field development

Force fields generated from the CHARMM General Force Field (CGenFF)^43^ using the paramchem interface (https://cgenff.paramchem.org) for the phosphodiester-linked **2** and the corresponding 1,2,3-triazole-linked **3** and carbamate-linked **4** derivatives (Fig. 2) were optimised using the GAAMP method^44^. The partial atomic charges (see Supplementary Fig. S1 and Table S1) and identified “soft” dihedrals, which were not already represented in the CHARMM and CGenFF force fields or that experienced a significant penalty score from paramchem, were optimised (see Supplementary Fig. S2).

### Molecular dynamics simulations

All MD simulations were prepared using VMD version 1.9.2^45^. An overview of all simulations is given in Supplementary Table S2. Simulation of human ST6Gal I (PDB id: 4JS2)–inhibitor complexes with the reported phosphodiester-linked compound **2** and the proposed 1,2,3-triazole and carbamate-linked derivatives (**3** and **4** respectively; Fig. 2) were undertaken to determine if a carbamate or 1,2,3-triazole is a suitable replacement for the phosphodiester linker used in previously reported inhibitors. The initial structure for the ST6Gal I–inhibitor complexes was taken from previously reported docking studies^40^. Additionally, simulations of each inhibitor in a water box were also performed to determine the effect of linker flexibility. Prior to simulation being undertaken PROPKA 3.1^46^ was used to predict the protonation state of ST6Gal I residues and ionisable ligand groups at pH 7.0.

Preparation of each simulation involved solvating the system in a box of water molecules, which extended approximately 9 Å from the surface of the protein, resulting in each system being solvated in an 82 Å × 82 Å × 82 Å cubic TIP3P^47^ water box. Each box was neutralised with counter ions of Na^+^ and Cl^−^, with the salt (NaCl) concentration set to 0.15 mol/L. Simulations were carried out using the NAMD 2.9 package^48^. The protein was represented with the non-polarisable CHARMM PARAM36 force field^49^. The force field for each ligand were those optimised using the GAAMP method^44^. All systems were simulated in periodic boundary conditions using the Langevin algorithm for maintaining the temperature at 298.15 K, and the Langevin Piston Nose–Hoover method^50,51^ for maintaining a constant pressure of 1.0 bar. The electrostatic interactions were calculated using the Particle Mesh Ewald method^52^. The van der Waals forces were treated with a cut-off of 12 Å and a smoothening function between 10 and 12 Å. All of the covalent bonds involving hydrogen were kept rigid with the RATTLE algorithm^53^. The integration time step was set to 1.0 fs, 2.0 fs and 4.0 fs for bonded, non-bonded and long-range electrostatics, respectively.

Equilibrium simulations for all human ST6Gal I-inhibitor complex systems were initially undertaken. During these simulations, a harmonic restraint was placed upon all Cα atoms of human ST6Gal I, with a decreasing force constant from 32.0 to 1.0 kcal/mol/Å^2^ over 10 ns. This decrease was achieved gradually by halving the force constant for each respective 1 ns interval until a force constant of 1.0 kcal/mol/Å^2^ was obtained (i.e. 32.0, 16.0 … 1.0 kcal/mol/Å^2^). A force constant of 1.0 kcal/mol/Å^2^ was then applied for the remainder of the 10 ns thermal equilibration. The simulations were further continued for 90 ns without any restraints (see Supplementary Table S2). The inhibitor only simulations (see Supplementary Table S2) were performed for 20 ns with a harmonic restraint placed upon one atom of the corresponding ligand, with a force constant of 32 kcal/mol/Å^2^. To perform trajectory analysis, snapshots were saved every ps (i.e. every 1,000 steps). Replicates of each simulation from different starting coordinates were generated by starting each simulation with three different initial minimisation steps (i.e. 2,000, 2,500 and 3,000 steps) and initiated with different velocities. In total, 2.2 μs of MD simulations were performed.

### Simulation trajectory analysis

To monitor the global properties of the structural evolution during MD simulations, the atomic positional root-mean-square deviations (RMSD) with respect to a reference structure and the atomic positional root-mean-square fluctuations (RMSF) were calculated using VMD^45^.

Hydrogen bond interactions were analysed according to a geometric criterion. A hydrogen bond was defined by a minimum donor–hydrogen–acceptor angle of 120° and a maximum heavy atom distance of 3 Å. Hydrophobic contacts were also analysed according to a geometric criterion of a maximum distance of 4 Å between hydrophobic atom pairs. To identify these interactions CHARMM version 38a1^54^ was utilised.

The quasi-harmonic approximation^55–57^ was used to examine the loss of configurational entropy of each ligand upon binding to human ST6Gal I. This analysis was performed, using CHARMM version 38a1^54^, for trajectories encompassing 11–100 ns (11–55 ns and 56–100 ns were also performed to monitor convergence; See Supplementary Table S3) of each ligand in complex with human ST6Gal I and trajectories encompassing 1–20 ns (1–10 ns and 11–20 ns were also performed to monitor convergence; See Supplementary Table S3) of the ligand simulations. Prior to the analysis, the Cartesian coordinates of the ligands were aligned after least-squares fit superposition of the heavy atoms of all configurations onto the initial structure, to eliminate the overall translation and rotation. The change in configurational entropy (ΔS_conf_) was determined based on the following relationship:

1 $${{\rm{\Delta }}{\rm{S}}}_{{\rm{conf}}}={{\rm{S}}}_{({\rm{complex}})}-{{\rm{S}}}_{({\rm{free}})}.$$

Using the structures obtained from the MD simulations, conformational cluster analysis was performed for the ligands using MMTSB tools^58^. Clusters were identified using k-means clustering with an RMSD threshold for ligand heavy atoms of 1.6 Å. For each simulation, cluster analysis was based on combined trajectories of every 10 ps of triplicate simulations. This resulted in 27,000 structures used in the analysis of the complexed simulations and 6,000 structures for the ligand only simulations.

### Free energy perturbation calculations

All free-energy perturbation (FEP) calculations described herein were carried out using the NAMD 2.10 package^48^. The simulation parameters were as per the MD simulation method described above. Depending on the calculation the starting structure was taken from the ST6Gal I-inhibitor simulations of the *(R)*-diastereomers of either the phosphodiester-linked lead compound ***(R)*** **-2** or the carbamate-linked derivative ***(R)*** **-4** and temperature was maintained at either 288.15 K or 298.15 K (see Supplementary Table S2).

**Figure 3:**
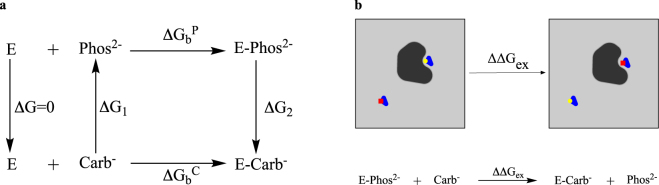
The thermodynamic cycle used (**a**) and a schematic representation of the perturbation performed (**b**) in the phosphodiester to carbamate FEP calculations. Phos and the yellow circle represent the phosphodiester-linked lead compound *(R)*-2, Carb and the red square represent the carbamate-linked derivative *(R)*-4, and E represents human ST6Gal I.

Each calculation utilised a dual ligand approach, where a ligand is mutated to an analogue in the human ST6Gal I binding site and the reverse mutation is performed simultaneously on the analogue in bulk water in the same system (Fig. 3). This approach has been undertaken to minimise potential electrostatic finite-size effects posed by the change in charge state when the *(R)*-diastereomer of the phosphodiester-linked lead compound **2** (net charge of -2e) is perturbed to the *(R)*-diastereomer of either the 1,2,3-triazole- **3** or carbamate-linked **4** derivatives (net charge of -1e) (Fig. 3)^59,60^. In this approach, the overall charge of the system remains constant. This allows for the reduction of errors arising from the large solvation energy of charged ligands and potential artefacts from the use of periodic boundary conditions. A similar approach has been successfully employed to handle changes in charge state in several different biomolecular systems^61^.

Using the dual ligand approach, the change in the binding free energy due to linker mutation with perturbation from phosphodiester to carbamate as an example can be expressed as (Fig. 3)

2 $$\begin{array}{c}{{\rm{\Delta }}{\rm{\Delta }}G}_{{\rm{b}}}={{\rm{\Delta }}{\rm{G}}}_{{\rm{b}}}^{{\rm{C}}}\mbox{--}{{\rm{\Delta }}{\rm{G}}}_{{\rm{b}}}^{{\rm{P}}}\\ \quad \quad \quad =\,{{\rm{\Delta }}{\rm{G}}}_{2}\mbox{--}{{\rm{\Delta }}{\rm{G}}}_{1}\\ \quad \quad \quad =\,{{\rm{\Delta }}{\rm{\Delta }}{\rm{G}}}_{{\rm{ex}}}\\ \quad \quad \quad =\,{{\rm{\Sigma }}}_{i}{{\rm{\Delta }}{\rm{\Delta }}{\rm{G}}}_{{\rm{ex}},i}\end{array}$$

In the FEP method, one introduces a hybrid Hamiltonian, *H*(λ) = (1 − λ)*H*_0_ + λ*H*_1_, where *H*_0_ represents the Hamiltonian for the initial state and *H*_1_ for the final state. The interval between 0 and 1 is divided into *n* subintervals (λ_*i*_, where *i* = 1, …, *n* – 1), and for each subinterval the free energy difference is calculated from the ensemble average, ΔΔG_ex,*i*_ = −*k*_*B*_T ln < exp[−(*H*(λ_*i*+1_) − *H*(λ_*i*_))/*k*_*B*_T > _λ*i*_. The free energy difference between initial and final states is obtained from the sum, ΔΔG = Σ_*i*_ ΔΔG_ex,*i*_. Alchemical transformations were carried out with 20 uniformly distributed λ values between 0 and 1 (0.0, 0.05, 0.1, …, 0.9, 0.95, 1.0), with the transformation simulated in reverse directions beginning from the 0.×5 λ values (e.g. 0.0 ← 0.05 → 0.1). To prevent the occurrence of singularities at values of λ where an atom disappears/appears, a soft-core potential with α = 4.0 Å^2^ was used to reshape the Lennard-Jones potential into a form devoid of singularity when λ approaches either 0 or 1^62,63^.

Each λ window underwent initial 5000 energy minimisation steps followed by 1 ns equilibration simulation to provide starting coordinates. Each window was then subsequently simulated for multiple ns with a harmonic restraint placed on the centre of mass of ST6Gal I. A harmonic restraint was also applied to one atom from the ligand in the bulk. These restraints were applied to prevent association of the ligand in the bulk and ST6Gal I and had no effects on the calculated binding free energy difference. Both restraints used a force constant of 32 kcal/mol/Å^2^. In total 1.02 μs of FEP simulations were performed.

## Results and Discussion

### 1,2,3-Triazole- and carbamate-linked *(R)-* and *(S)-*diastereoisomers maintain consistent binding site contacts with human ST6Gal I when compared to their phosphodiester derivative

Hydrogen bond and hydrophobic contact analysis of phosphodiester- and carbamate-linked *(R)*-diastereomers has previously been published^40^. These simulations were performed using ligand force fields generated using the general AMBER force field (GAFF)^64^ and with human ST6Gal I represented with the non-polarisable CHARMM PARAM36 force field^49^. In this work, a force field parameterisation compatible with the CHARMM PARAM36 force field was carried out for the ligands. We have undertaken simulation of both the *(R)-* and *(S)-*diastereoisomers of the phosphodiester- (**2**), 1,2,3-triazole- (**3**) and carbamate-linked (**4**) derivatives (Fig. 2) in complex with human ST6Gal I with the newly developed force field.

The MD simulations of each complex showed a stable trajectory with the positional RMSD of backbone Cα atoms being 1.5–2.0 Å for each simulation (see Supplementary Figs S3–S8). RMSFs for the Cα atoms, were generally consistent across all simulations (see Supplementary Figs S3–S8). Larger RMSF values, across each simulation, were observed for the flexible loop between Tyr355 and Tyr369. Large RMSF values were also observed for the regions between Gln235—Asn250 and Ser260—Ile295, which comprise the portion of the active site that is expected to interact with the sialyl acceptor. This can most likely be attributed to the phenoxy being within the proposed sialyl acceptor site. Across all simulations each ligand remained tightly associated with the human ST6Gal I active site. However, there was some variation in ligand position within this active site, which can be seen in the RMSD values that correspond to the heavy atoms of each ligand (see Supplementary Figs S3–S8). These fluctuations can be attributed to the *m*-phenoxy substituent, which depending upon the simulation either rotated to maintain contacts with the sialyl acceptor site or only weakly interacted with peripheral residues of this pocket, resulting in positional fluctuation.

**Figure 4:**
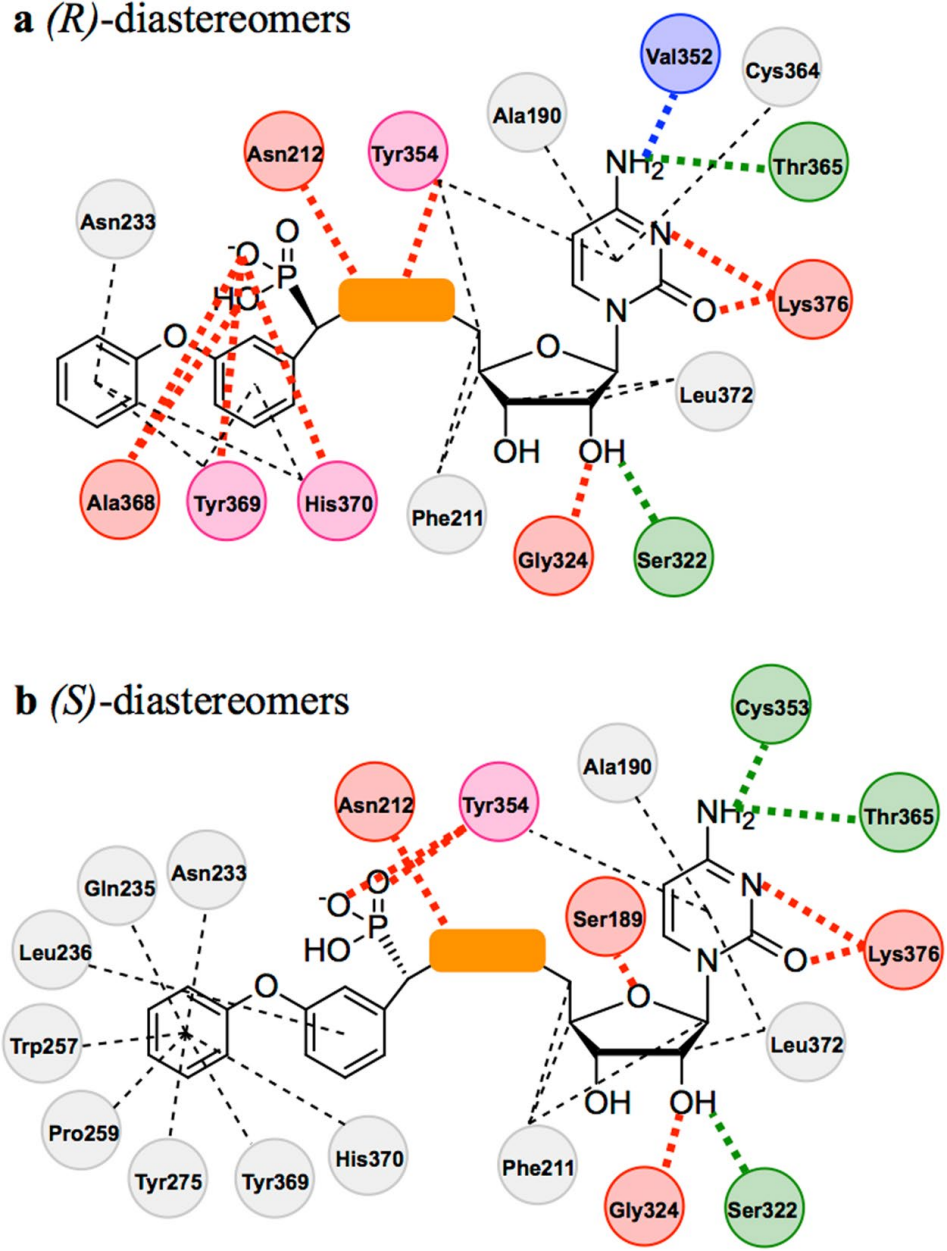
Schematic representation of conserved hydrogen bonds and hydrophobic contacts observed in the *(R)-*diastereomer- (**a**) and *(S)*-diastereomer-ST6Gal I complex (**b**) simulations. The consistent components of each inhibitor are shown as chemical structures. The orange box represents the different linkers. Interacting human ST6Gal I residues are represented as circles which are colour coded based on interaction type as follows: Red = hydrogen bond donor; Green = hydrogen bond acceptor; Blue = water bridged hydrogen bond; Grey = hydrophobic contact; Pink = hydrogen bond and hydrophobic contact. Dashed lines indicate hydrophobic contacts and dotted lines represent the hydrogen bonds shown in Supplementary Tables S4–S15.

Interactions in the human ST6Gal I-ligand complexes analysed were characterised by monitoring the hydrogen bonds and hydrophobic contacts (see Supplementary Tables S4–S15). Each simulation revealed similar interactions with the ST6Gal I active site, which have been schematically illustrated in Fig. 4.

The cytidine moiety of each simulation’s respective compound maintained the hydrogen bonding interactions that were observed in the human ST6Gal I crystal structure^25^. An additional interaction observed in all simulations was Lys376 acting as a hydrogen bond donor to the pyridine like nitrogen of the cytosine ring. There was also Ser189 acting as a hydrogen bond donor with the oxygen embedded within the furan ring in the simulations of the *(S)*-diastereomers (Fig. 4b). The hydrophobic contacts that were observed were most significant with the cytosine ring, which was shown to interact with Ala190 and have a proposed π-stacking interaction with Tyr354. The ribose ring was generally shown to have hydrophobic interactions with Phe211 and Leu372. It should be noted that in the simulations of the *(R)*-diastereomers only (Fig. 4a), a water bridge interaction between the free amine of the cytosine ring and the backbone carbonyl of Val352 was also observed.

In addition to the CMP moiety, each compound had two other conserved regions, the phosphonate and the Neu5Ac mimic. For the simulation involving the *(R)*-diastereomers the phosphonate was shown to hydrogen bond with His370 in all simulations and, Ala368 and Tyr369 in all simulations except TC1 (see Supplementary Table S8). The phosphonate in the simulations of the *(S)*-diastereomers occupied a different orientation within the ST6Gal I active site and was shown to hydrogen bond with Tyr354 in all simulations. This difference between the diastereomers is to be expected as the phosphonate is a substituent at the only varied chiral centre. This could also be an explanation to the higher level of potency observed for ***(R)*** **-2** compared to ***(S)*** **-2** when tested against rat α-2,6 ST^29^, which has been shown to have a high level of homology to the human equivalent^23^.

The Neu5Ac mimic consists of two components, the aryl ring and its *m*-phenoxy substituent. The *m*-phenoxy substituent for the majority of the simulations was shown to be in, or oriented towards, the proposed sialyl acceptor site through observed hydrophobic contacts with Asn233, Gln235, Trp257, Pro259, Tyr275 and Tyr369. This was most evident for the *(S)*-diastereomers. More variation in this group’s position was observed for the *(R)*-diastereomers, with the phenoxy generally orientated to interact only with the residues on the periphery of the pocket, being Asn233, Tyr369 and His370. Notably of the three different classes of linker observed, it was the 1,2,3-triazole that showed both the *(R)*- and *(S)*-diastereomers with the same *m*-phenoxy interactions (see Supplementary Tables S9 and S11), suggesting the added rigidity of the linker favours direction towards the proposed sialyl acceptor site. This is an important finding, as this site is expected to vary between the different ST subtypes, particularly those with different sialyl acceptors, and thus presents a potential route to the development of selective human ST6Gal I inhibitors.

The aryl ring of the Neu5Ac mimic was shown to have limited interactions with human ST6Gal I, with the *(R)*-diastereomers showing hydrophobic contacts with Tyr369 and His370 and the *(S)*-diastereomers with Leu236. Despite being limited these interactions are nevertheless important in directing the *m*-phenoxy towards the proposed sialyl acceptor site. This is clearly highlighted in the CL2 simulations (see Supplementary Table S2) where this aryl ring is shown to interact with Cys353, Tyr354 and Cys364 in a binding mode that does not see the *m*-phenoxy within the proposed sialyl acceptor site.

The only component of the potential ST inhibitors examined that varied was the group that links the CMP moiety to the Neu5Ac mimic. Consistent interactions were observed when comparing the phosphodiester linker to the 1,2,3-triazole- and carbamate linkers. In the simulations of the *(R)*-diastereomers, hydrogen bonds of the linkers with Asn212 and Tyr354 were observed. In the simulations of the *(S)*-diastereomers hydrogen bonds of the linkers with Asn212 were observed.

Analysis of the hydrogen bond and hydrophobic contacts of both the *(R)*- and *(S)*-diastereomers of the three different classes of compounds, has demonstrated that no matter which linker is used, key interactions of cytidine and the Neu5Ac mimic with the human ST6Gal I active site are able to be maintained. These results confirm our previous findings^40^ and provide further evidence that a carbamate or a 1,2,3-triazole may be a suitable isosteric replacement for the phosphodiester linkers that have been used in reported ST inhibitors.

### Binding free energy calculations confirm 1,2,3-triazole and carbamate as isosteres of the phosphodiester linker

To build upon the results observed in the MD simulations on the thermodynamic level, FEP calculations were performed to study the relative binding affinities for compounds with three different linkers. These simulations involved the alchemical transformations of the phosphodiester-linked ***(R)*** **-2** to either the 1,2,3-triazole ***(R)*** **-3** or carbamate-linked ***(R)*** **-4** derivatives. To close the thermodynamic cycle in order to monitor the convergence, an additional transformation of the carbamate-linked ***(R)*** **-4** to the 1,2,3-triazole-linked ***(R)*** **-3** was also performed. The results of the FEP calculations are summarised in Table 1.

**Table 1:** Difference in binding free energy to human ST6Gal I of proposed ST inhibitors.

Alchemical Transformation	ΔΔG_b_		ΔΔH_b_^c^	−TΔΔS_b_^c^
	288 K^a^	298 K^b^	—	—
2 → 4	−1.1 ± 0.8	−1.7 ± 0.4	10 ± 30	−20 ± 30
2 → 3	−0.3 ± 0.3	−1.4 ± 0.9	40 ± 30	−40 ± 30
4 → 3	−0.7 ± 1.0	−0.5 ± 0.3	−10 ± 30	10 ± 30

Running average of ΔΔG_b_ (kcal/mol) calculated at ^a^288.15 K and ^b^298.15 K, error reported is the SEM. ^c^The ΔΔH_b_ (kcal/mol) and −TΔΔS_b_ (kcal/mol) are calculated through finite-temperature difference from the ΔΔG_b_ values for both the simulations carried out at 288.15 K and 298.15 K. The values reported are those for 298.15 K, error reported is the SEM.

The ΔΔG_b_ values observed for the 288 K and 298 K calculations form a thermodynamic cycle which closes within a reasonable error of −1.5 kcal/mol and −0.8 kcal/mol respectively. These calculations indicate that there is only a slight difference in the free energy of binding between the carbamate- and 1,2,3-triazole-linked derivatives, with the 1,2,3-triazole favoured. It was also demonstrated that when the phosphodiester linker of the lead compound is perturbed to either a carbamate or a 1,2,3-triazole, a slightly more favourable binding to ST6Gal I is observed (−1.7 kcal/mol and −1.4 kcal/mol respectively at 298.15 K), with the carbamate being slightly more preferential.

This result is surprising as the charge of the phosphodiester linker has been previously thought to be important for binding, but to what extent is currently unknown^29,32,34,35,38^. It is expected that replacing the charged phosphodiester group with a neutral isostere could potentially result in the loss of favourable interactions with the binding site that are mediated by this charge. For example, in the MD simulations the phosphodiester linker was involved in three additional water mediated interactions that were not observed in those of the carbamate- and 1,2,3-triazole-linked derivatives (see Supplementary Tables S4, S8, S12). Therefore, one would expect an impact on the binding free energy (ΔG_b_), particularly through enthalpy (ΔH_b_), if the charged phosphodiester linker were to be replaced. Based on the relatively similar binding free energies for compounds with three different linkers, we hypothesise that the more rigid isosteric linkers might achieve comparable binding affinities through a favourable entropy compensation.

The error analyses for the FEP simulations were carried out by calculated the standard error of the mean (SEM) based on each ns simulation segment (Table 1) and all simulations converged with an error of <1.0 kcal/mol. RMSD of both protein backbone and complexed ligand were also monitored. RMSDs for Cα of ST6Gal I converged between 1.5 Å and 2.0 Å indicating that no significant change in the structure of the protein backbone occurred as one linker was perturbed to another. Across all simulations each ligand remained tightly associated with the ST6Gal I active site, with RMSD values that fluctuated between 1.0 Å and 3.0 Å observed. As with the MD simulations discussed above, this fluctuation was influenced by the *m*-phenoxy substituent, with it remaining fixed in the sialyl acceptor site for the lower RMSDs observed and with it orientated towards the sialyl acceptor site but not constantly located within it when larger RMSDs were observed.

### Relative rigidity of the 1,2,3-triazole and carbamate linkers compensate for the enthalpic penalty that results from replacing the phosphodiester

To test our enthalpy-entropy compensation hypothesis proposed based on the FEP calculations, we calculated ΔΔH_b_ and ΔΔS_b_ from the ΔΔG_b_ values observed at two different temperatures (Table 1)^65^. From this analysis, we were able to demonstrate that the replacement of the charged phosphodiester linker with either a neutral carbamate or 1,2,3-triazole indeed results in an enthalpic penalty (a positive ΔΔH_b_). In both cases this penalty was compensated for by a favourable entropy (a negative ΔΔS_b_). This supports our hypothesis of the resulting molecular rigidity created by the inclusion of a carbamate or 1,2,3-triazole in place of a phosphodiester compensates for the enthalpic penalty. For the perturbation from the carbamate to the 1,2,3-triazole, the opposite relationship was observed where changing to a triazole was enthalpically favourable which compensated for an entropic penalty.

The effect of the entropic component of binding free energy to human ST6Gal I has also been demonstrated through quasi-harmonic analysis of the trajectories obtained from the MD simulations performed (Table 2). This analysis has shown that for both the *(R)-* and *(S)-*diastereomers, the phosphodiester-linked lead compound **2** has a larger configurational entropy penalty upon binding to human ST6Gal I when compared to the 1,2,3-triazole- **3** and carbamate-linked **4** derivatives. Though the quasi-harmonic analysis only captures the configurational entropy of the ligands upon binding, while neglecting contributions from other factors, the relatively large difference observed supports the entropic compensation that was captured in the component analysis of the ΔΔG_b_ values (Table 1). It can also be seen that there is comparatively a very small difference (that is within error) between the −TΔS_conf_ values for the carbamate and 1,2,3-triazole (Table 2). However, the 1,2,3-triazole does exhibit a slightly larger value for both the *(R)-* and *(S)-*diastereomers supporting the entropic penalty observed when a carbamate is perturbed to a 1,2,3-triazole (Table 1).

**Table 2:** Configurational entropy change upon binding of phosphodiester-linked (2), 1,2,3-triazole-linked (3) and carbamate-linked (4) derivatives to human ST6Gal I.

Cpd	ΔS_conf_ (kcal/mol/K)^a^	−TΔS_conf_ (kcal/mol)^b^
*(R)*-2	−0.125 ± 0.009	37 ± 3
*(S)*-2	−0.14 ± 0.02	41 ± 6
*(R)*-3	−0.08 ± 0.01	23 ± 4
*(S)*-3	−0.09 ± 0.01	27 ± 4
*(R)*-4	−0.067 ± 0.007	20 ± 2
*(S)*-4	−0.09 ± 0.02	25 ± 6

^a^Arithmetic mean ΔS_conf_ ± standard error of the mean (SEM) obtained from triplicate simulations of each compound. ^b^Arithmetic mean ΔS_conf_ multiplied by −298.15 K ± SEM obtained from triplicate simulations of each compound.

Comparing the diastereomers it can be seen while there are no major differences between the diastereomers, the *(S)-*diastereomer in all three cases has a slightly larger entropic penalty for binding to ST6Gal I. This result reflects the slight differences in the binding modes of the diastereomers observed during the analysis of the MD simulations.

Overall both the free energy component analysis and the quasi-harmonic analysis have demonstrated that the conformational restriction, and thus entropic favourability for binding, imparted by the 1,2,3-triazole and carbamate linkers when compared to the phosphodiester linker is enough to compensate the resulting enthalpic penalty. This result has provided a strong rationale for the use of these two groups as isosteres of the phosphodiester in the development of potential inhibitors of human ST6Gal I. Interestingly, while we have demonstrated the benefit of introducing conformational restriction to binding, there appears to be a limit to what can be tolerated. This is reflected in the carbamate being shown to be slightly more entropically favourable than the 1,2,3-triazole (Tables 1 and 2).

### Conformational cluster analysis demonstrates different linker flexibilities

Additional clustering analysis was performed for the ligand conformations in the human ST6Gal I-ligand complex and in the aqueous solution. The most dominant cluster for each complex simulation (see Supplementary Fig. S9) showed similar positions for the cytidine and phenoxy substituents for both the *(R)*- and *(S)*-diastereomers. This supports the findings of the analysis of the protein-ligand interactions (Fig. 4). The conformations of these structures also provide an indication of the differing levels of flexibility of each linker, with the conformation of the 1,2,3-triazole derivatives being much more rigid and elongated when compared to the phosphodiester- and carbamate-linked counterparts. However, this conformational difference is much more obvious in the distribution of cluster population obtained from the ligand in water box simulations (see Supplementary Fig. S10). The distribution for the *(R)*-diastereomers shows the phosphodiester-linked ligand samples a much larger conformational space than both its carbamate- and 1,2,3-triazole-linked counterparts, as evidenced by the number of clusters identified. The distribution for the *(S)*-diastereomers shows a similar trend, however the difference between the phosphodiester and carbamate derivatives is not as large. The increased flexibility of the phosphodiester derivatives compared to the 1,2,3-triazole- and carbamate-linked derivatives provides further evidence to support the enthalpy-entropy compensation hypothesis.

It is worth noting that though the binding free energy differences are converged within 1.0 kcal/mol, a proper error propagation significantly increases the uncertainties in the enthalpic component (ΔΔH_b_) and entropic component (−TΔΔS_b_) (Table 1). Nevertheless, our calculations have demonstrated that these three types of inhibitors with different linkers have similar potencies against human ST6Gal I. Complementary analyses based on binding entropy calculations, quasi-harmonic and conformational cluster analyses highlighted the potential role of entropy in the binding of transition state-based ST6Gal I inhibitors. Experimentally, we are synthesising both the 1,2,3-triazole- and carbamate-linked derivatives and their predicted potency will be validated with experimental inhibition rates and kinetic constants. The enthalpy-entropy compensation hypothesis can be then validated by examining the temperature-dependent data.

## Conclusion

MD simulations and FEP calculations were performed to investigate the replacement of the charged phosphodiester linker used in reported TS analogue ST inhibitors with a neutral isostere, such as a carbamate or a 1,2,3-triazole. MD simulations of the *(R)-* and *(S)-*diastereomers of three ligands, which only differed in the linker, complexed with human ST6Gal I or in a water box were performed. Hydrogen bonds and hydrophobic contacts of the ligands with human ST6Gal I were monitored over the course of the simulations and have demonstrated that no matter which linker is used, important interactions with the active site are able to be maintained. These results confirmed the findings of our previous work^40^, which was undertaken with a different set of force fields. Free energy perturbation calculations demonstrated that when the phosphodiester linker was perturbed to either a carbamate or a 1,2,3-triazole a slightly more favourable binding to human ST6Gal I was observed, with the carbamate being marginally more preferential. This result was a surprise considering the perceived importance of the phosphodiester linker. We rationalise this observation with a hypothesis of an enthalpy-entropy compensation, which is supported with free energy component analysis, quasi-harmonic and cluster analysis. These analyses demonstrated that the conformational restriction, and thus entropic favourability for binding, imparted by the 1,2,3-triazole and carbamate linkers when compared to the phosphodiester linker is enough to compensate the resulting enthalpic penalty. The results of these simulations have provided a strong rationale for the use of a carbamate or 1,2,3-triazole as an isostere of the phosphodiester. We are currently exploring both potential isosteres synthetically with the aim to develop novel inhibitors of human ST6Gal I, improve synthetic accessibility and address potential pharmacokinetic problems.

## Electronic supplementary material

Supplementary Information

## References

[1] Angata T, Varki A (2002). Chemical diversity in the sialic acids and related α-keto acids: An evolutionary perspective. Chem. Rev..

[2] Li Y, Chen X (2012). Sialic acid metabolism and sialyltransferases: Natural functions and applications. Appl. Microbiol. Biotechnol..

[3] Harduin-Lepers A (2001). The human sialyltransferase family. Biochimie.

[4] Varki A (1997). Sialic acids as ligands in recognition phenomena. FASEB J..

[5] Bull C, Stoel MA, den Brok MH, Adema GJ (2014). Sialic acids sweeten a tumor’s life. Cancer Res..

[6] Pinho SS, Reis CA (2015). Glycosylation in cancer: mechanisms and clinical implications. Nat Rev Cancer.

[7] Huang S, Day TW, Choi M-R, Safa AR (2009). Human β-galactoside α-2,3-sialyltransferase (ST3Gal III) attenuated Taxol-induced apoptosis in ovarian cancer cells by downregulating caspase-8 activity. Mol. Cell. Biochem..

[8] Szabo R, Skropeta D (2017). Advancement of Sialyltransferase Inhibitors: Therapeutic Challenges and Opportunities. Med. Res. Rev..

[9] Chen X, Varki A (2010). Advances in the biology and chemistry of sialic acids. ACS Chem. Biol..

[10] Lombard V, Golaconda Ramulu H, Drula E, Coutinho PM, Henrissat B (2014). The carbohydrate-active enzymes database (CAZy) in 2013. Nucleic Acids Res..

[11] Audry M (2011). Current trends in the structure-activity relationships of sialyltransferases. Glycobiology.

[12] Schultz MJ, Swindall AF, Bellis SL (2012). Regulation of the metastatic cell phenotype by sialylated glycans. Cancer Metastasis Rev..

[13] Schultz, M. J. *et al*. ST6Gal-I sialyltransferase confers cisplatin resistance in ovarian tumor cells. *J. Ovarian Res*. **6**, 10.1186/1757-2215-6-25 (2013). 10.1186/1757-2215-6-25 PMC3637436 23578204

[14] Cerami E (2012). The cBio Cancer Genomics Portal: An open platform for exploring multidimensional cancer genomics data. Cancer Discov..

[15] Swindall AF (2013). ST6Gal-I protein expression is upregulated in human epithelial tumors and correlates with stem cell markers in normal tissues and colon cancer cell lines. Cancer Res..

[16] Schultz MJ (2016). The Tumor-Associated Glycosyltransferase ST6Gal-I Regulates Stem Cell Transcription Factors and Confers a Cancer Stem Cell Phenotype. Cancer Res..

[17] Seales EC (2005). Hypersialylation of β 1 integrins, observed in colon adenocarcinoma, may contribute to cancer progression by up-regulating cell motility. Cancer Res..

[18] Shaikh FM (2008). Tumor cell migration and invasion are regulated by expression of variant integrin glycoforms. Exp. Cell Res..

[19] Swindall AF, Bellis SL (2011). Sialylation of the Fas death receptor by ST6Gal-I provides protection against Fas-mediated apoptosis in colon carcinoma cells. J. Biol. Chem..

[20] Liu Z (2011). ST6Gal-I regulates macrophage apoptosis via alpha2-6 sialylation of the TNFR1 death receptor. J. Biol. Chem..

[21] Britain CM, Dorsett KA, Bellis SL (2017). The Glycosyltransferase ST6Gal-I Protects Tumor Cells against Serum Growth Factor Withdrawal by Enhancing Survival Signaling and Proliferative Potential. J. Biol. Chem..

[22] Lairson LL, Henrissat B, Davies GJ, Withers SG (2008). Glycosyl transferases: Structures, functions, and mechanisms. Annu. Rev. Biochem..

[23] Meng L (2013). Enzymatic basis for N-glycan sialylation: Structure of rat α2,6-sialyltransferase (ST6GAL1) reveals conserved and unique features for glycan sialylation. J. Biol. Chem..

[24] Breton C, Fournel-Gigleux S, Palcic MM (2012). Recent structures, evolution and mechanisms of glycosyltransferases. Curr. Opin. Struct. Biol..

[25] Kuhn B (2013). The structure of human α-2,6-sialyltransferase reveals the binding mode of complex glycans. Acta Crystallogr. D.

[26] Jung KH, Schwörer R, Schmidt RR (2003). Sialyltransferase Inhibitors. Trends Glycosci. Glyc..

[27] Wang X, Zhang LH, Ye XS (2003). Recent development in the design of sialyltransferase inhibitors. Med. Res. Rev..

[28] Drinnan NB, Halliday J, Ramsdale T (2003). Inhibitors of sialyltransferases: potential roles in tumor growth and metastasis. Mini Rev. Med. Chem..

[29] Skropeta D, Schwörer R, Haag T, Schmidt RR (2004). Asymmetric synthesis and affinity of potent sialyltransferase inhibitors based on transition-state analogues. Glycoconjugate J..

[30] Schröder PN, Giannis A (1999). From substrate to transition state analogues: The first potent inhibitor of sialyltransferases. Angew. Chem. Int. Ed..

[31] Amann F, Schaub C, Müller B, Schmidt RR (1998). New potent sialyltransferase inhibitors - Synthesis of donor and of transition-state analogues of sialyl donor CMP-Neu5Ac. Chem. Eur. J..

[32] Schwörer R, Schmidt RR (2002). Efficient sialyltransferase inhibitors based on glycosides of N-acetylglucosamine. J. Am. Chem. Soc..

[33] Müller B, Schaub C, Schmidt RR (1998). Efficient sialyltransferase inhibitors based on transition-state analogues of the sialyl donor. Angew. Chem. Int. Ed..

[34] Li W, Niu Y, Xiong D-C, Cao X, Ye X-S (2015). Highly Substituted Cyclopentane–CMP Conjugates as Potent Sialyltransferase Inhibitors. J. Med. Chem..

[35] Guo J, Li W, Xue W, Ye X-S (2017). Transition State-Based Sialyltransferase Inhibitors: Mimicking Oxocarbenium Ion by Simple Amide. J. Med. Chem..

[36] Volkers G (2015). Structure of human ST8SiaIII sialyltransferase provides insight into cell-surface polysialylation. Nat. Struct. Mol. Biol..

[37] Skropeta D, Schwörer R, Schmidt RR (2003). Stereoselective synthesis of phosphoramidate α(2-6)sialyltransferase transition-state analogue inhibitors. Bioorg. Med. Chem. Lett..

[38] Kumar R (2013). Sialyltransferase inhibitors: Consideration of molecular shape and charge/hydrophobic interactions. Carbohydr. Res..

[39] Rye CS, Baell JB (2005). Phosphate isosteres in medicinal chemistry. Curr. Med. Chem..

[40] Montgomery A, Szabo R, Skropeta D, Yu H (2016). Computational characterisation of the interactions between human ST6Gal I and transition-state analogue inhibitors: insights for inhibitor design. J. Mol. Recogn.

[41] Montgomery, A. P., Xiao, K., Wang, X., Skropeta, D. & Yu, H. in *Advances in Protein Chemistry and Structural Biology* Vol. 109 (ed Karabencheva-Christova Tatyana) 25–76 (Academic Press, 2017). 10.1016/bs.apcsb.2017.04.003 28683920

[42] Van Gunsteren WF (2006). Biomolecular modeling: Goals, problems, perspectives. Angew. Chem., Int. Ed..

[43] Vanommeslaeghe K (2010). CHARMM general force field: A force field for drug-like molecules compatible with the CHARMM all-atom additive biological force fields. J. Comput. Chem..

[44] Huang L, Roux B (2013). Automated force field parameterization for nonpolarizable and polarizable atomic models based on ab initio target data. J. Chem. Theory Comput..

[45] Humphrey W, Dalke A, Schulten K (1996). VMD: Visual molecular dynamics. J. Mol. Graph. Model..

[46] Søndergaard CR, Olsson MHM, Rostkowski M, Jensen JH (2011). Improved treatment of ligands and coupling effects in empirical calculation and rationalization of pKa values. J. Chem. Theory Comput..

[47] Jorgensen WL, Chandrasekhar J, Madura JD, Impey RW, Klein ML (1983). Comparison of simple potential functions for simulating liquid water. J. Chem. Phys.

[48] Phillips JC (2005). Scalable molecular dynamics with NAMD. J. Comput. Chem..

[49] MacKerell AD (1998). All-Atom Empirical Potential for Molecular Modeling and Dynamics Studies of Proteins. J. Phys. Chem. B.

[50] Martyna GJ, Tobias DJ, Klein ML (1994). Constant pressure molecular dynamics algorithms. J. Chem. Phys.

[51] Feller SE, Zhang Y, Pastor RW, Brooks BR (1995). Constant pressure molecular dynamics simulation: The Langevin piston method. J. Chem. Phys.

[52] Darden T, York D, Pedersen L (1993). Particle mesh Ewald: An N·log(N) method for Ewald sums in large systems. J. Chem. Phys.

[53] Andersen HC (1983). Rattle: A “velocity” version of the shake algorithm for molecular dynamics calculations. J. Comput. Phys.

[54] Brooks BR (2009). CHARMM: The biomolecular simulation program. J. Comput. Chem..

[55] Karplus M (1981). Method for estimating the configurational entropy of macromolecules. Macromolecules.

[56] Andricioaei I, Karplus M (2001). On the calculation of entropy from covariance matrices of the atomic fluctuations. J. Chem. Phys.

[57] Schäfer H, Mark AE, Gunsteren WFv (2000). Absolute entropies from molecular dynamics simulation trajectories.. J. Chem. Phys.

[58] Feig M, Karanicolas J, Brooks CL (2004). MMTSB Tool Set: enhanced sampling and multiscale modeling methods for applications in structural biology. J. Mol. Graphics Modell..

[59] Rocklin GJ, Mobley DL, Dill KA, Hünenberger PH (2013). Calculating the binding free energies of charged species based on explicit-solvent simulations employing lattice-sum methods: An accurate correction scheme for electrostatic finite-size effects. J. Chem. Phys.

[60] Reif MM, Oostenbrink C (2014). Net charge changes in the calculation of relative ligand-binding free energies via classical atomistic molecular dynamics simulation. J. Comput. Chem..

[61] Heinzelmann G, Baştuğ T, Kuyucak S (2011). Free Energy Simulations of Ligand Binding to the Aspartate Transporter GltPh. Biophys. J..

[62] Zacharias M, Straatsma TP, McCammon JA (1994). Separation‐shifted scaling, a new scaling method for Lennard‐Jones interactions in thermodynamic integration. J. Chem. Phys.

[63] Beutler TC, Mark AE, van Schaik RC, Gerber PR, van Gunsteren WF (1994). Avoiding singularities and numerical instabilities in free energy calculations based on molecular simulations. Chem. Phys. Lett..

[64] Wang J, Wolf RM, Caldwell JW, Kollman PA, Case DA (2004). Development and testing of a general amber force field. J. Comput. Chem..

[65] Peter C, Oostenbrink C, van Dorp A, van Gunsteren WF (2004). Estimating entropies from molecular dynamics simulations. J. Chem. Phys.

