# Supplementary material for: Transition state-based ST6Gal I inhibitors: Mimicking the phosphodiester linkage with a triazole or carbamate through an enthalpy-entropy compensation
Source: Sci Rep. 2017 Oct 31;7:14428. doi: 10.1038/s41598-017-14560-0 (PMC5663928; doi:10.1038/s41598-017-14560-0)
Supplement: Supplementary file 1 — Supplementary Information [file 41598_2017_14560_MOESM1_ESM.pdf]

Transition state-based ST6Gal I inhibitors: Mimicking the phosphodiester linkage with a triazole or carbamate through an enthalpy-entropy compensation

Andrew P. Montgomery[a], Danielle Skropeta[a, b] & Haibo Yu[a, b, c *]

[a] School of Chemistry, Faculty of Science, Medicine and Health, University of Wollongong, Wollongong NSW 2522, Australia

[b] Centre for Medical and Molecular Bioscience, University of Wollongong, Wollongong NSW 2522, Australia

[c] Illawarra Health and Medical Research Institute, Wollongong, NSW 2522, Australia

[*]**Corresponding Author:** hyu@uow.edu.au

Table of Contents

Force Field Optimisation **S4**

Figure S1. *The atom designation used during GAAMP optimisation of the phosphodiester- **(2)**, triazole- **(3)** and carbamate-linked **(4)** derivatives force fields. The black region on each compound represents atoms where the atom charge was fixed to be consistent with the value observed after GAAMP optimisation of the phosphodiester-linked compound. Red regions represent components where partial charge were independently optimised by GAAMP.* *S4*

Table S1: *GAAMP optimisation of MM charges* *S4*

Figure S2. (a) *Comparison of QM, GAAMP and CGenFF dihedral energy profiles for phosphodiester-linked derivative **2**. ϕ_1 represents the dihedral O9-P1-C9-O7 and ϕ_2 represents the dihedral C10-C9-O7-P. **(b)** Comparison of QM, GAAMP and CGenFF dihedral energy profiles for triazole-linked derivative **3**. ϕ_1 represents the dihedral O1-C7-C8-N3 and ϕ_2 represents the dihedral N5-C10-C11-C12. **(c)** Comparison of QM, GAAMP and CGenFF dihedral energy profiles for carbamate-linked derivative **4**. ϕ_1 represents the dihedral O1-C7-C8-N3 and ϕ_2 represents the dihedral O5-C10-P-O8.* *S5*

Overview of Simulations **S6**

Table S2: *Overview of Simulations* *S6*

Monitoring of Quasi-Harmonic Approximation Convergence **S7**

Table S3. *Monitoring convergence of configurational entropy change upon binding of the phosphodiester-linked **2**, 1,2,3 triazole-linked **3** and carbamate-linked **4** derivatives to human ST6Gal I.* *S7*

RMSD and RMSF of PC1 Simulations **S8**

Figure S3. (A) *RMSD of the Cα atoms across the 90 ns of free simulation. (B) RMSD of the heavy atoms of the phosphodiester-linked compound **(R)-2** across the 90 ns of free simulation. (C) RMSF of the Cα atoms across the 90 ns of free simulation. RMSD and RMSF were calculated after superposition to the starting structure with respect to the position of Cα.* *S8*

RMSD and RMSF of PC2 Simulations **S9**

Figure S4. (a) *RMSD of the Cα atoms across the 90 ns of free simulation. **(b)** RMSD of the heavy atoms of the phosphodiester-linked compound **(S)-2** across the 90 ns of free simulation (large fluctuations in ligand RMSD during the PC1-2000 simulation can be attributed to the phenoxy group not being locked into a single conformation). **(c)** RMSF of the Cα atoms across the 90 ns of free simulation. RMSD and RMSF were calculated after superposition to the starting structure with respect to the position of Cα.* *S9*

RMSD and RMSF of TC1 Simulations **S10**

Figure S5. (a) *RMSD of the Cα atoms across the 90 ns of free simulation. **(b)** RMSD of the heavy atoms of the triazole-linked **(R)-3** across the 90 ns of free simulation (the large increase in ligand RMSD during the TC1-2000 and -3000 simulations can be attributed to a rotation of the phosphonate and phenoxy group and a phenoxy positional shift respectively. In both cases the phenoxy substituent remained within the expected pocket). **(c)** RMSF of the Cα atoms across the 90 ns of free simulation. RMSD and RMSF were calculated after superposition to the starting structure with respect to the position of Cα* *S10*

RMSD and RMSF of TC2 Simulations **S11**

Figure S6. (a) *RMSD of the Cα atoms across the 90 ns of free simulation. **(b)** RMSD of the heavy atoms of the triazole-linked **(S)-3** across the 90 ns of free simulation. **(c)** RMSF of the Cα atoms across the 90 ns of free simulation. RMSD and RMSF were calculated after superposition to the starting structure with respect to the position of Cα.* *S11*

RMSD and RMSF of CC1 Simulations **S12**

Figure S7. (a) *RMSD of the Cα atoms across the 90 ns of free simulation. **(b)** RMSD of the heavy atoms of the carbamate-linked **(R)-4** across the 90 ns of free simulation. **(c)** RMSF of the Cα atoms across the 90 ns of free simulation. RMSD and RMSF were calculated after superposition to the starting structure with respect to the position of Cα.* *S12*

RMSD and RMSF of CC2 Simulations **S13**

Figure S8. (a) *RMSD of the Cα atoms across the 90 ns of free simulation. **(b)** RMSD of the heavy atoms of the carbamate-linked **(S)-4** across the 90 ns of free simulation (large RMSD values, in particular for the CC2-2000 simulation, can be attributed to a slight phenoxy rotation from the starting structure to maintain interactions with Tyr369). **(c)** RMSF of the Cα atoms across the 90 ns of free simulation.* *S13*

RMSD and RMSF were calculated after superposition to the starting structure with respect to the position of Cα.

Non-Bonded Interactions of Phosphodiester-Linked Inhibitor *(R)*-2 **S14**

Table S4. *Hydrogen bonding and water bridge interactions observed for the PC1 simulations* *S14*

Table S5. *Hydrophobic contacts observed for the PC1 simulations* *S15*

Non-Bonded Interactions of Phosphodiester-Linked Inhibitor *(S)*-2 **S16**

Table S6. *Hydrogen bonding and water bridge interactions observed for the PC2 simulations* *S16*

Table S7. *Hydrophobic contacts observed for the PC2 simulations* *S17*

Non-Bonded Interactions of Triazole-Linked Inhibitor *(R)*-3 **S18**

Table S8. *Hydrogen bonding and water bridge interactions observed for the TC1 simulations* *S18*

Table S9. *Hydrophobic contacts observed for the TC1 simulations* *S19*

Non-Bonded Interactions of Triazole-Linked Inhibitor *(S)*-3 **S20**

Table S10. *Hydrogen bonding and water bridge interactions observed for the TC2 simulations* *S20*

Table S11. *Hydrophobic contacts observed for the TC2 simulations* *S21*

Non-Bonded Interactions of Carbamate-Linked Inhibitor *(R)*-4 **S22**

Table S12. *Hydrogen bonding and water bridge interactions observed for the CC1 simulations* *S22*

Table S13. *Hydrophobic contacts observed for the CC1 simulations* *S23*

Non-Bonded Interactions of Carbamate-Linked Inhibitor *(S)*-4 **S24**

Table S14. *Hydrogen bonding and water bridge interactions observed for the CC2 simulations* *S24*

Table S15. *Hydrophobic contacts observed for the CC2 simulations* *S25*

Cluster Analysis of Complexed MD Simulations **S26**

Figure S9. *The most populated clusters of each respective ligand from the human ST6Gal I-ligand complex simulations. Clusters were generated using combined trajectories that represented every 10 ps of the triplicate simulations during their 90 ns production runs (i.e. 27,000 structures in total per simulation type). The clusters shown represent the following percentage of simulation time: PC1 68%; TC1 49%; CC1 31%; PC2 67%; TC2 42%; CC2 67%.* *S26*

Cluster Analysis of Ligand MD Simulations **S27**

Figure S10. *The distribution of cluster population for the (R)-* ***(A)*** *and (S)-inhibitors* ***(B)*** *in water box MD simulations. Clusters were generated using combined trajectories that represented every 10 ps of the triplicate simulations (i.e. 6,000 structures in total per simulation type).* *S27*

Force Field Optimisation

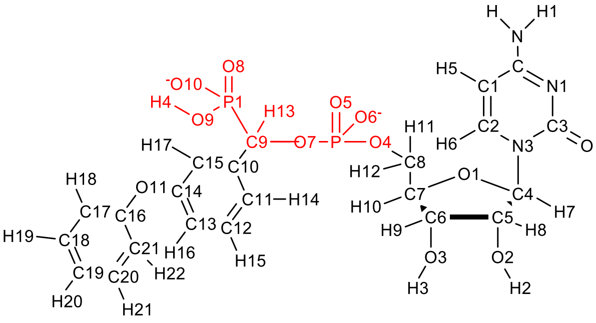

2

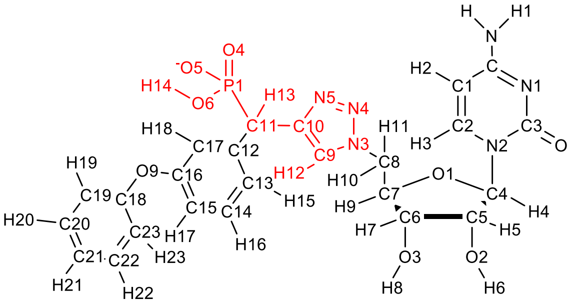

3

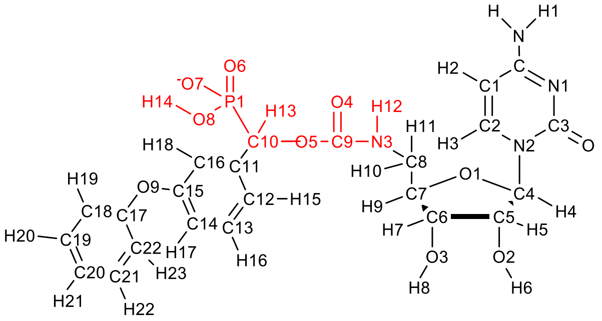

4

Figure S1. The atom designation used during GAAMP optimisation of the phosphodiester- (**2**), triazole- (**3**) and carbamate-linked (**4**) derivatives force fields. The black region on each compound represents atoms where the atom charge was fixed to be consistent with the value observed after GAAMP optimisation of the phosphodiester-linked compound. Red regions represent components where partial charge were independently optimised by GAAMP. The final parameters for these three inhibitors can be accessed at https://doi.org/10.6084/m9.figshare.5372788.v1 The rational for us to develop the new force field using cGen/FF/GAAMP was to generate a consistent set of force field parameters for protein and ligands, as previous simulations were carried out with the ligand force field generated by Antechamber/GAFF which is not consistent with the adopted protein force field (CHARMM).

Table S1: GAAMP optimisation of MM charges[a]

Compound	χ^2 [b]	$\chi_{ESP}^2 \pm$ SE [c]	$\chi_{Emin}^2 \pm$ SE [c]	$\chi_{Rmin}^2 \pm$ SE [c]	$\chi_{CG}^2 \pm$ SE [c]
**2**	1.60	0.398 ± 0.006	1 ± 2	0.02 ± 0.05	0.0 ± 0.1
**3**	2.23	1.80 ± 0.01	0.3 ± 0.9	0.1 ± 0.1	0.0 ± 0.2
**4**	1.98	1.04 ± 0.01	1 ± 1	0.1 ± 0.1	0.0 ± 0.2

[a] Optimisation occurs at the HF/6-31G* level without basis set superposition error correction. [b] $\chi^2 = \chi_{ESP}^2 + \chi_{wat\_int}^2 + \chi_{CG}^2$; where $\chi_{wat\_int}^2 = \chi_{Emin}^2 + \chi_{Rmin}^2$. χ^2 is the objective function used in the optimisation procedure of the MM charges. It is written as the sum of three terms: the objective functions for the electrostatic potential (χ_{ESP}^2), compound-water interactions ($\chi_{wat\_int}^2$) and the restraints on reference charges (χ_{CG}^2). [c] SE = Standard error.

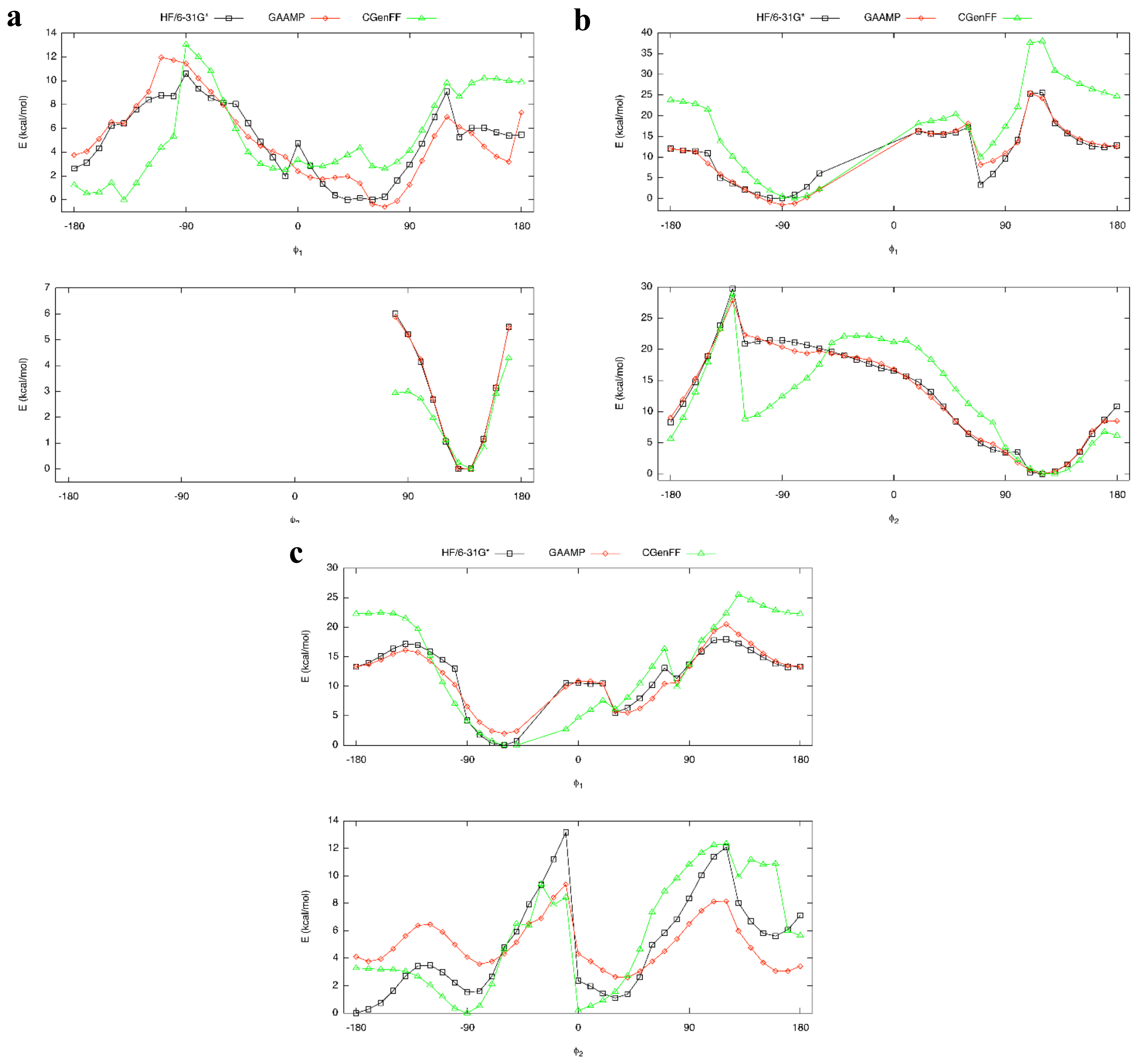

Figure S2. (a) Comparison of QM, GAAMP and CGenFF dihedral energy profiles for phosphodiester-linked derivative **2**. ϕ_1 represents the dihedral O9-P1-C9-O7 and ϕ_2 represents the dihedral C10-C9-O7-P. **(b)** Comparison of QM, GAAMP and CGenFF dihedral energy profiles for triazole-linked derivative **3**. ϕ_1 represents the dihedral O1-C7-C8-N3 and ϕ_2 represents the dihedral N5-C10-C11-C12. **(c)** Comparison of QM, GAAMP and CGenFF dihedral energy profiles for carbamate-linked derivative **4**. ϕ_1 represents the dihedral O1-C7-C8-N3 and ϕ_2 represents the dihedral O5-C10-P-O8.

Overview of Simulations

Table S2: Overview of Simulations

Name	Ligand	Equilibration (ns)	Free Simulation (ns)
**ST6Gal I—inhibitor complex MD simulations**[a,b]			
PC1 (×3)	Phosphodiester—linked inhibitor ***(R)*-2**	10	90
PC2 (×3)	Phosphodiester—linked inhibitor ***(S)*-2**	10	90
TC1(×3)	1,2,3-Triazole—linked inhibitor ***(R)-3***	10	90
TC2 (×3)	1,2,3-Triazole—linked inhibitor ***(S)*-3**	10	90
CC1 (×3)	Carbamate—linked inhibitor ***(R)*-4**	10	90
CC2 (×3)	Carbamate—linked inhibitor ***(S)*-4**	10	90
**Inhibitor in water box MD simulations**[b,c]			
PL1 (×3)	Phosphodiester—linked inhibitor ***(R)*-2**	-	20
PL2 (×3)	Phosphodiester—linked inhibitor ***(S)*-2**	-	20
TL1 (×3)	1,2,3-Triazole—linked inhibitor ***(R)-3***	-	20
TL2 (×3)	1,2,3-Triazole—linked inhibitor ***(S)*-3**	-	20
CL1 (×3)	Carbamate—linked inhibitor ***(R)*-4**	-	20
CL2 (×3)	Carbamate—linked inhibitor ***(S)*-4**	-	20
**Free energy perturbation simulations**[d,e]			
PL1 → CL1 288 K	Phosphodiester—linked inhibitor ***(R)*-2** to Carbamate—linked inhibitor ***(R)*-4**	-	140
PL1 → CL1 298 K	Phosphodiester—linked inhibitor ***(R)*-2** to Carbamate—linked inhibitor ***(R)*-4**	-	200
PL1 → TL1 288 K	Phosphodiester—linked inhibitor ***(R)*-2** to 1,2,3-Triazole—linked inhibitor ***(R)-3***	-	140
PL1 → TL1 298 K	Phosphodiester—linked inhibitor ***(R)*-2** to 1,2,3-Triazole—linked inhibitor ***(R)-3***	-	200
CL1 → TL1 288 K	Carbamate—linked inhibitor ***(R)*-4** to 1,2,3-Triazole—linked inhibitor ***(R)-3***	-	140
CL1 → TL1 298 K	Carbamate—linked inhibitor ***(R)*-4** to 1,2,3-Triazole—linked inhibitor ***(R)-3***	-	200

[a]The systems consist of approximately 49,000 atoms with a box size of 82 Å × 82 Å × 82 Å.

[b]Each simulation was replicated three times by generating different starting coordinates using three different initial minimisation steps (2,000, 2,500 and 3,000 steps) and initial velocities.

[c]The systems consist of approximately 50,000 atoms with a box size of 82 Å × 82 Å × 82 Å.

[d]Each system consists of 20 uniformly distributed λ values between 0 and 1 (0.0, 0.05, 0.1, … , 0.9, 0.95, 1.0) with the transformation simulated in reverse directions beginning from the 0.×5 λ values (e.g. 0.0 ← 0.05 → 0.1).

[e]The systems consist of approximately 58,000 atoms with a box size of 86 Å × 86 Å × 86 Å.

Monitoring of Quasi-harmonic Approximation Convergence

Table S3. Monitoring convergence of configurational entropy change upon binding of the phosphodiester-linked **2**, 1,2,3-triazole-linked **3** and carbamate-linked **4** derivatives to human ST6Gal I.

Compound	$-T\Delta S_{conf}$ (kcal/mol)[a]				
	11—100 ns/ 1—20 ns[b]	11—55 ns/ 1—20 ns[c]	56—100 ns/ 1—20 ns[d]	11—55 ns/ 1—10 ns[e]	56—100 ns/ 11—20 ns[f]
***(R)*-2**	37 ± 3	37 ± 3	40 ± 2	33 ± 2	34 ± 4
***(S)*-2**	41 ± 6	40 ± 6	42 ± 4	36 ± 6	35 ± 5
***(R)*-3**	23 ± 4	29 ± 2	28 ± 4	27 ± 2	20 ± 5
***(S)*-3**	27 ± 4	32 ± 1	31 ± 5	27 ± 2	27 ± 5
***(R)*-4**	20 ± 2	22.1 ± 0.7	21 ± 3	17 ± 3	17 ± 3
***(S)*-4**	25 ± 6	25 ± 6	31 ± 6	24 ± 5	26 ± 5

[a]Arithmetic mean ΔS_{conf} multiplied by -298.15 K ± SEM obtained from triplicate simulations of each compound. [b]Calculated using the MD trajectories of the 11th to 100th ns of ST6Gal-inhibitor complex and 1st to 20th ns of ligand in a water box; [c]11th to 55th ns of ST6Gal I-inhibitor complex and 1st to 20th ns of ligand a water box; [d]56th to 100th ns of ST6Gal I-inhibitor complex and 1st to 20th ns of ligand in a water box; [e]1st to 55th ns of ST6Gal I-inhibitor complex and 1st to 10th ns of ligand a water box; [f]56th to 100th ns of ST6Gal I-inhibitor complex and 11th to 20th ns of ligand a water box.

RMSD and RMSF of PC1 Simulations

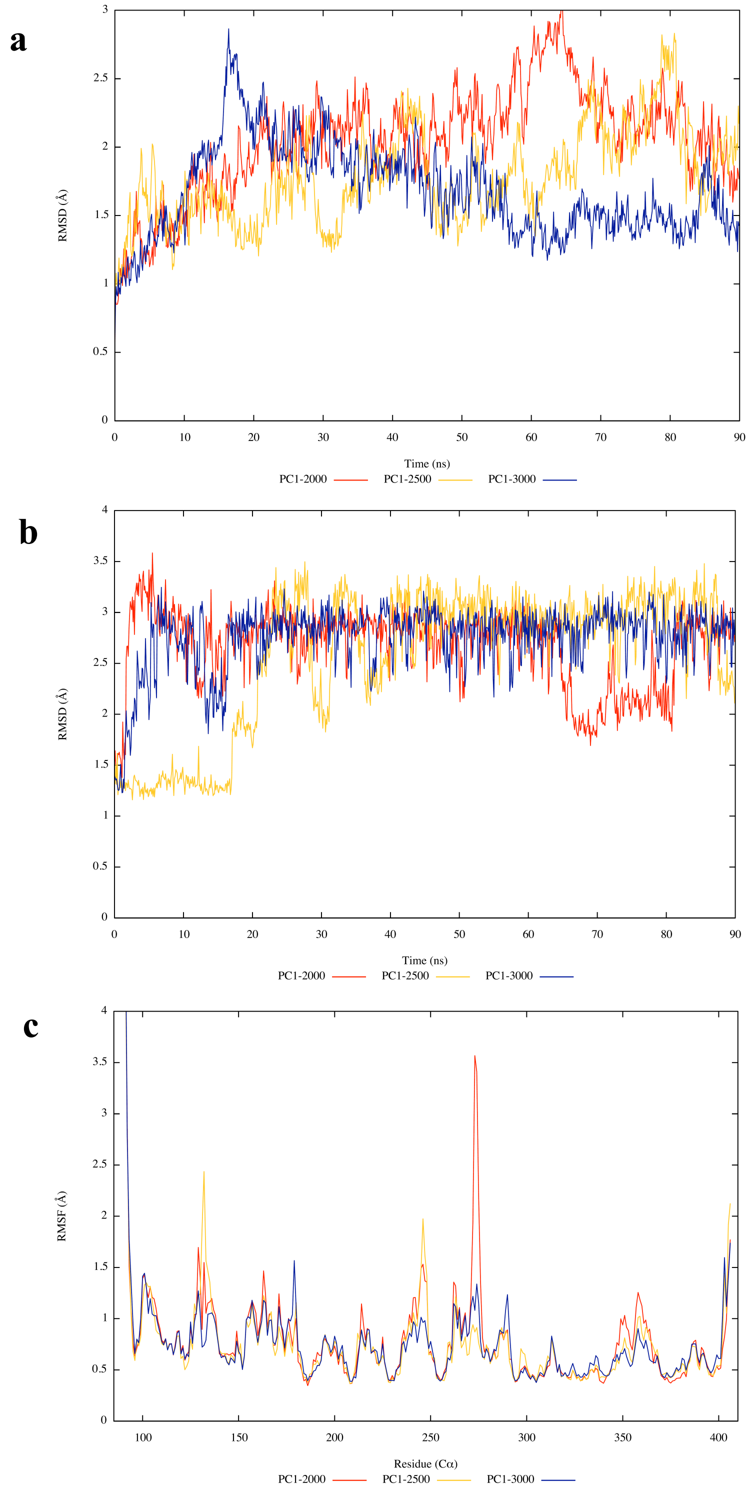

Figure S3. (a) RMSD of the Cα atoms across the 90 ns of free simulation. **(b)** RMSD of the heavy atoms of the phosphodiester-linked compound ***(R)*-2** across the 90 ns of free simulation. **(c)** RMSF of the Cα atoms across the 90 ns of free simulation. RMSD and RMSF were calculated after superposition to the starting structure with respect to the position of Cα.

RMSD and RMSF of PC2 Simulations

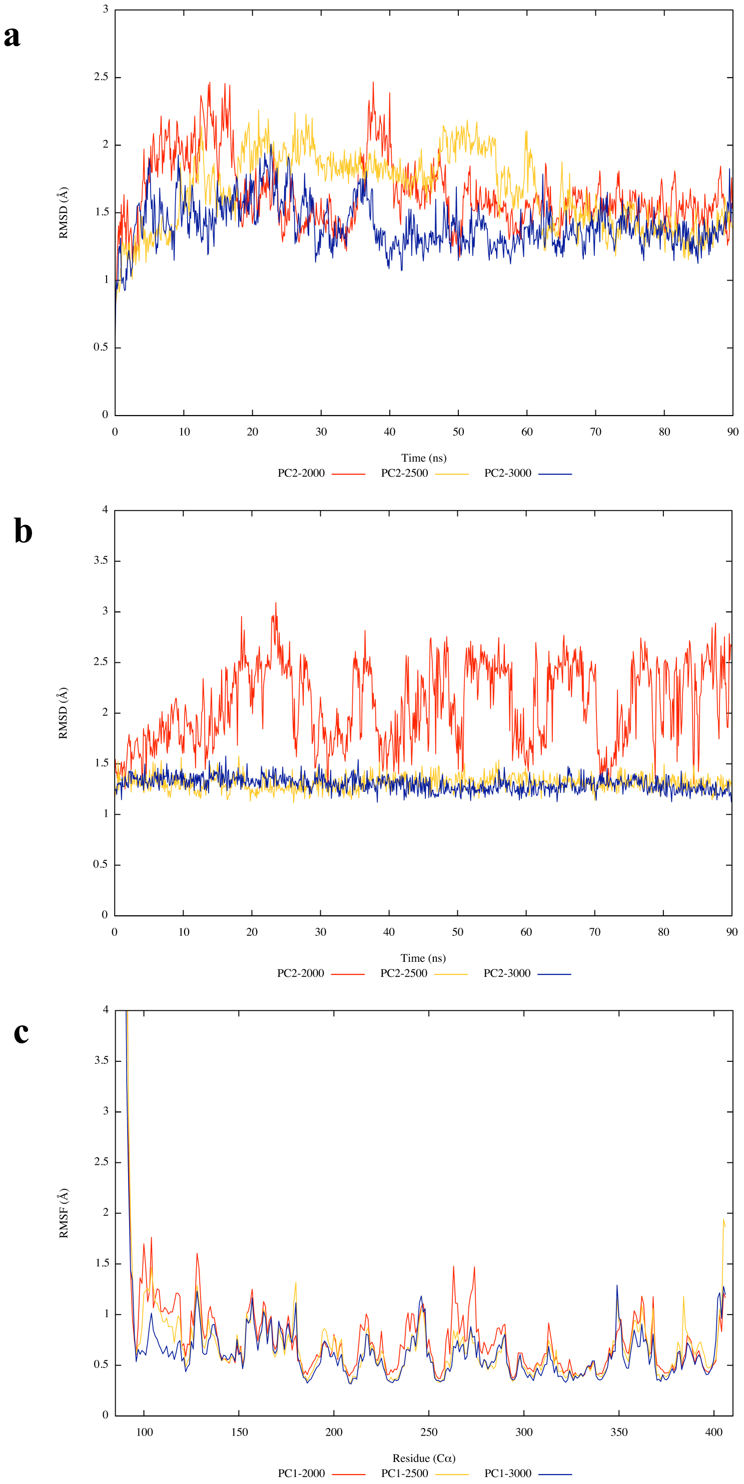

Figure S4. (a) RMSD of the Cα atoms across the 90 ns of free simulation. **(b)** RMSD of the heavy atoms of the phosphodiester-linked compound ***(S)*-2** across the 90 ns of free simulation (large fluctuations in ligand RMSD during the PC1-2000 simulation can be attributed to the phenoxy group not being locked into a single conformation). **(c)** RMSF of the Cα atoms across the 90 ns of free simulation. RMSD and RMSF were calculated after superposition to the starting structure with respect to the position of Cα.

RMSD and RMSF of TC1 Simulations

a

b

c

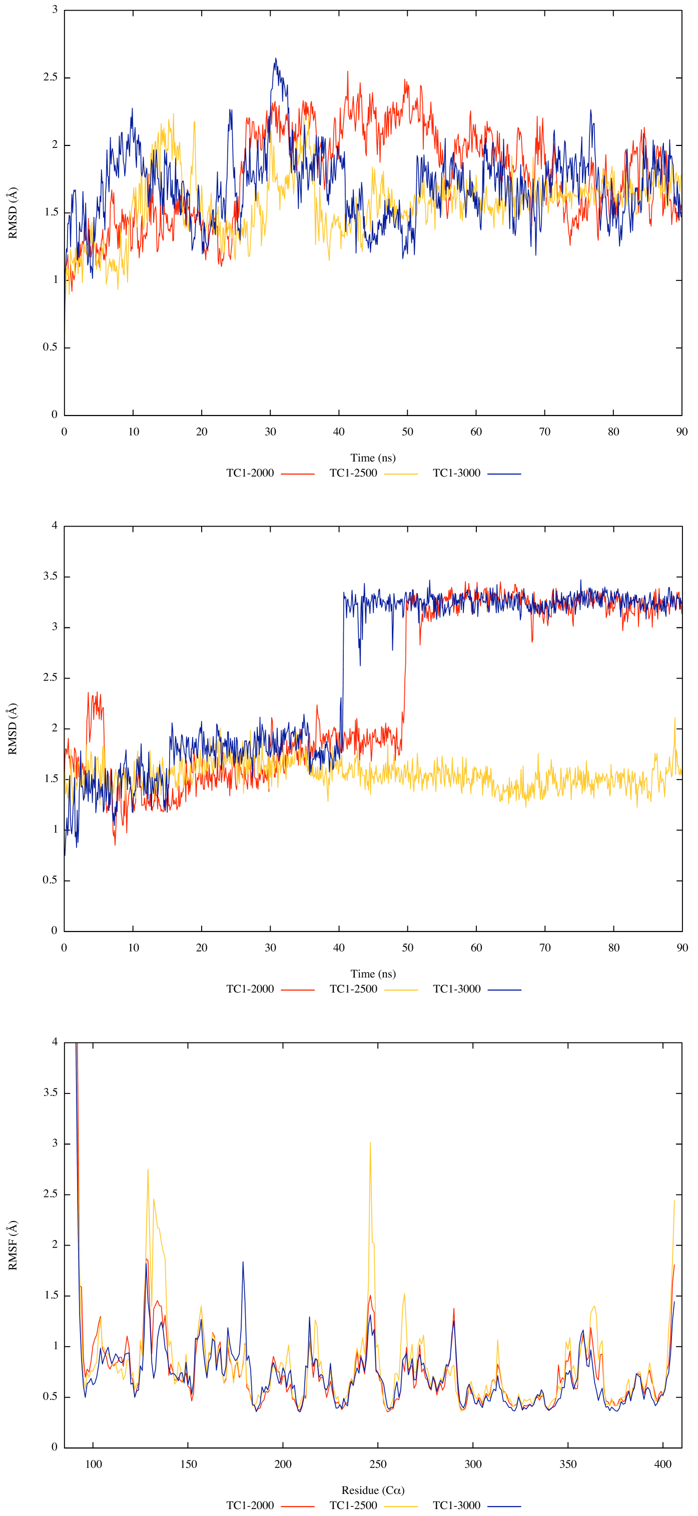

Figure S5. (a) RMSD of the Cα atoms across the 90 ns of free simulation. **(b)** RMSD of the heavy atoms of the triazole-linked ***(R)*-3** across the 90 ns of free simulation (the large increase in ligand RMSD during the TC1-2000 and -3000 simulations can be attributed to a rotation of the phosphonate and phenoxy group and a phenoxy positional shift respectively. In both cases the phenoxy substituent remained within the expected pocket). **(c)** RMSF of the Cα atoms across the 90 ns of free simulation. RMSD and RMSF were calculated after superposition to the starting structure with respect to the position of Cα.

RMSD and RMSF of TC2 Simulations

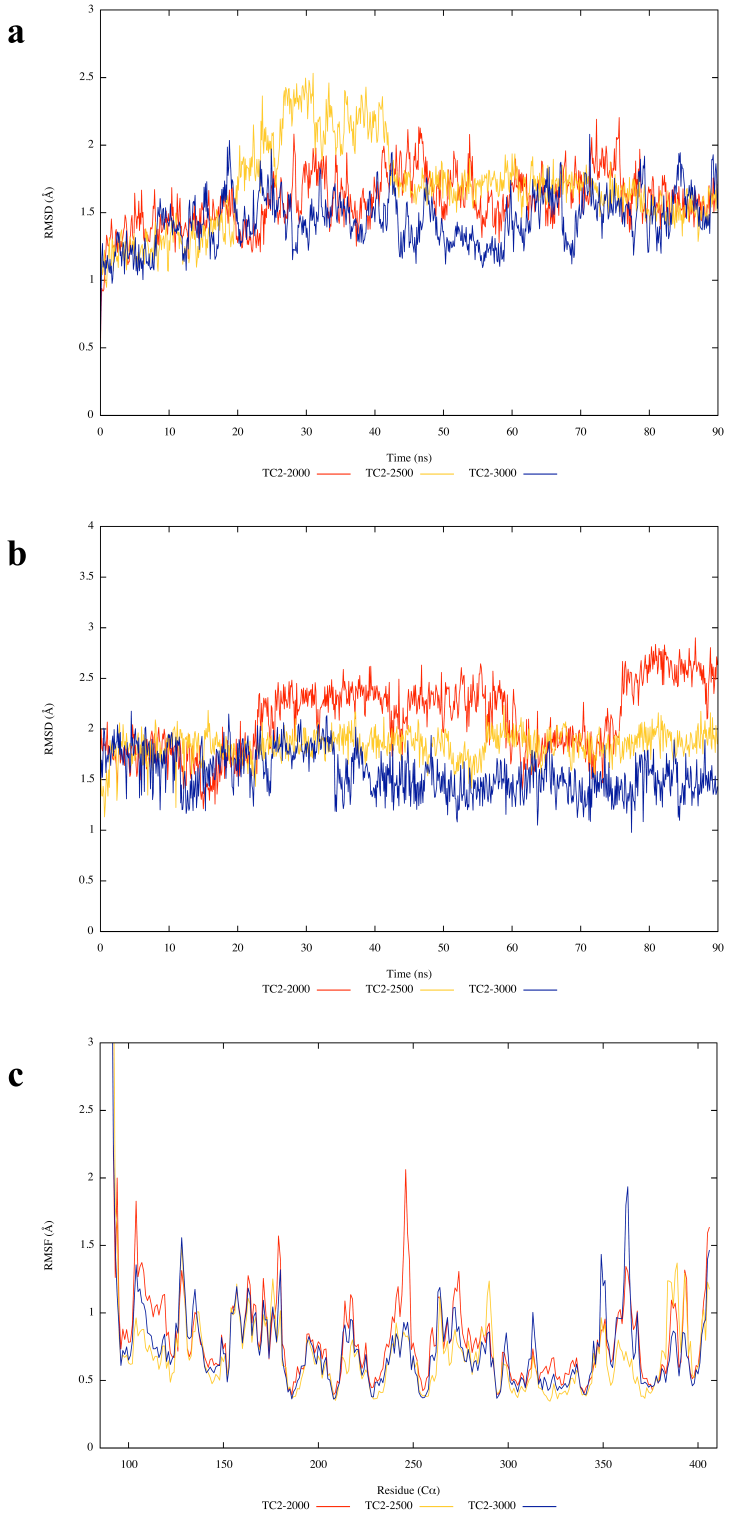

Figure S6. (a) RMSD of the Cα atoms across the 90 ns of free simulation. **(b)** RMSD of the heavy atoms of the triazole-linked ***(S)*-3** across the 90 ns of free simulation. **(c)** RMSF of the Cα atoms across the 90 ns of free simulation. RMSD and RMSF were calculated after superposition to the starting structure with respect to the position of Cα.

RMSD and RMSF of CC1 Simulations

a

b

c

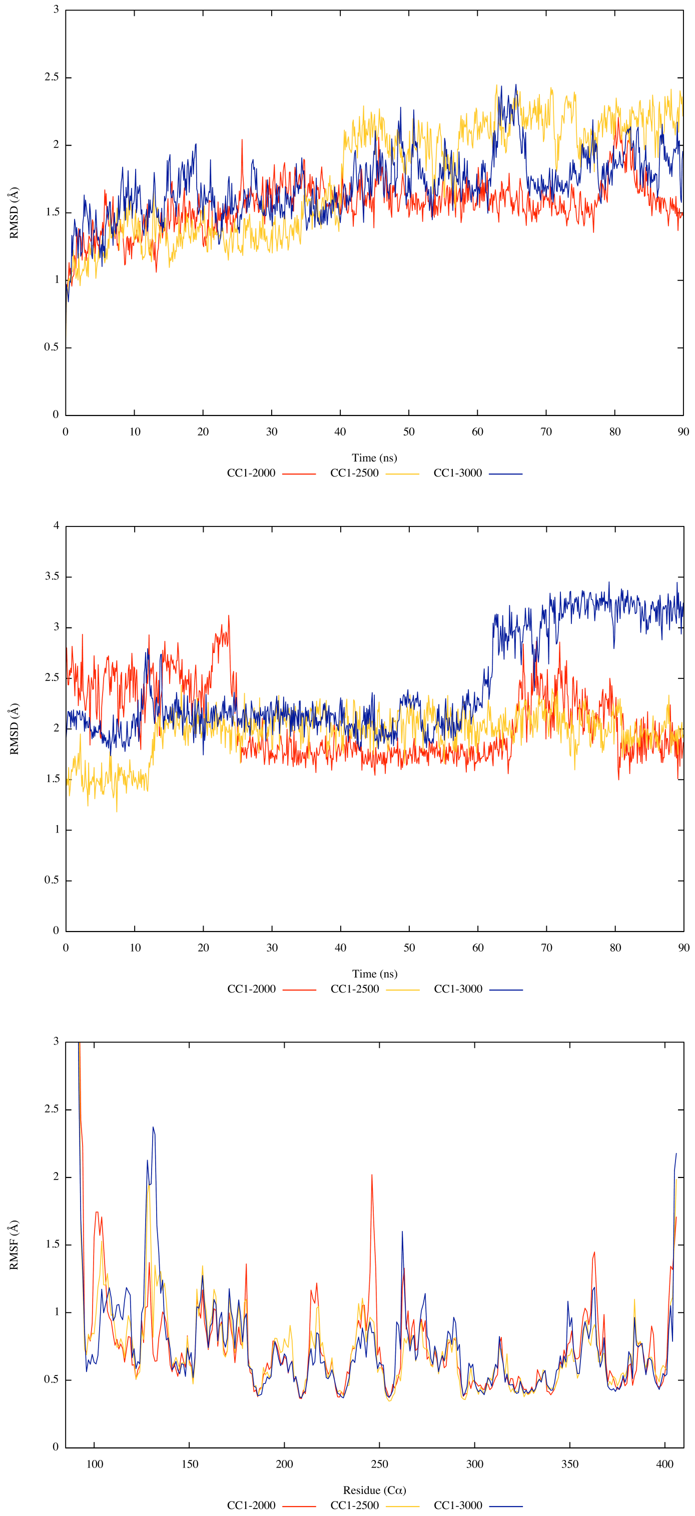

Figure S7. (a) RMSD of the Cα atoms across the 90 ns of free simulation. **(b)** RMSD of the heavy atoms of the carbamate-linked ***(R)*-4** across the 90 ns of free simulation. **(c)** RMSF of the Cα atoms across the 90 ns of free simulation. RMSD and RMSF were calculated after superposition to the starting structure with respect to the position of Cα.

RMSD and RMSF of CC2 Simulations

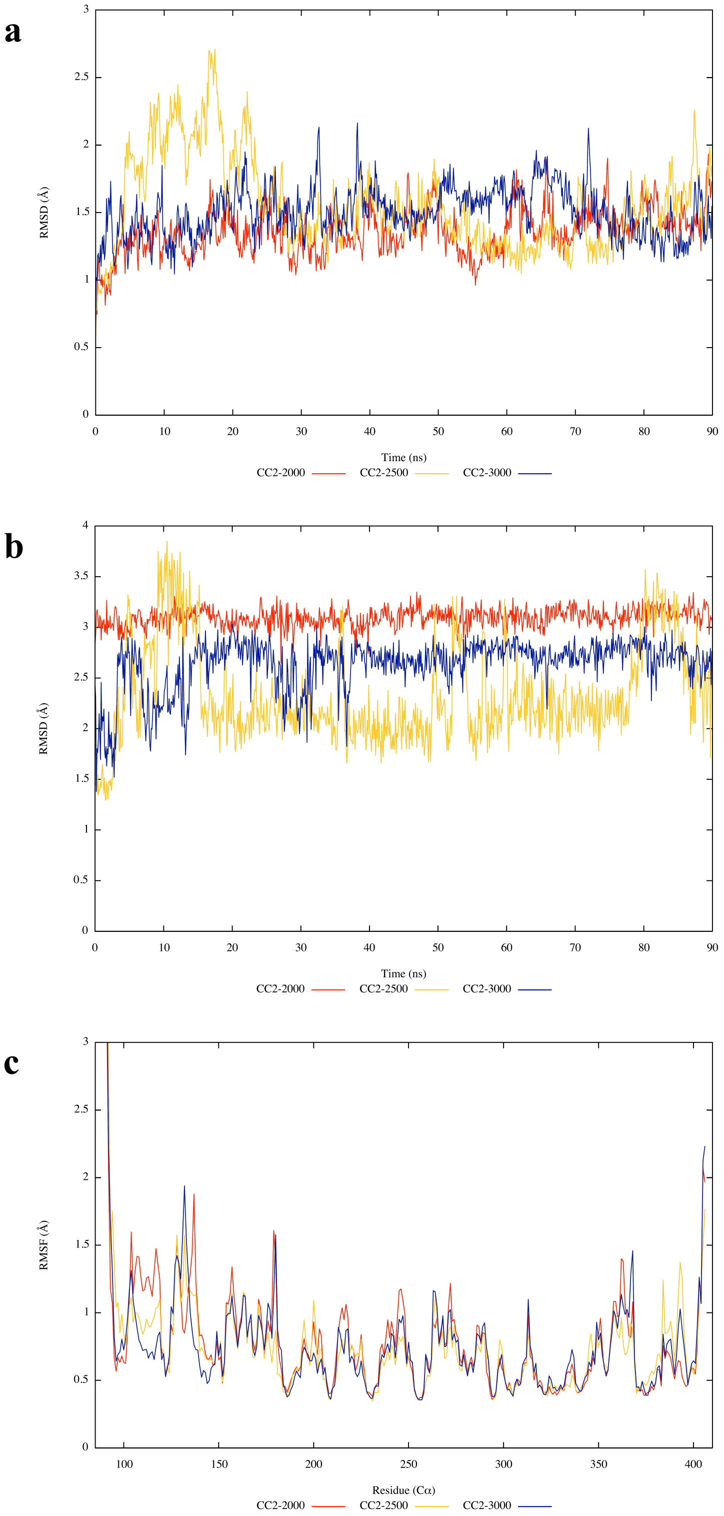

Figure S8. (a) RMSD of the Cα atoms across the 90 ns of free simulation. **(b)** RMSD of the heavy atoms of the carbamate-linked ***(S)*-4** across the 90 ns of free simulation (large RMSD values, in particular for the CC2-2000 simulation, can be attributed to a slight phenoxy rotation from the starting structure to maintain interactions with Tyr369). **(c)** RMSF of the Cα atoms across the 90 ns of free simulation. RMSD and RMSF were calculated after superposition to the starting structure with respect to the position of Cα.

Non-Bonded Interactions of Phosphodiester-Linked Inhibitor *(R)*-2

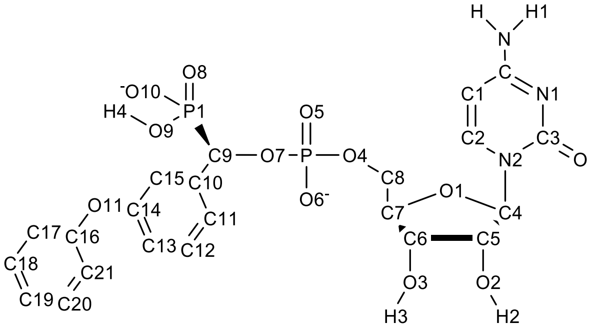

Table S4. Hydrogen bonding and water bridge interactions observed for the PC1 simulations

Simulation	Hydrogen Bonds	Occupancy (%)	Hydrogen Bonds	Occupancy (%)
PC1[a,b]	$HZ1_{Lys376}$-O_{PC1}	30	HN_{Ala368}-$O9_{PC1}$	26
	$HZ2_{Lys376}$-O_{PC1}	31	HN_{Ala368}-$O10_{PC1}$	37
	$HZ3_{Lys376}$-O_{PC1}	29	HN_{Tyr369}-$O10_{PC1}$	25
	NH_{Gly324}-$O2_{PC1}$	18	HN_{His370}-$O10_{PC1}$	26
	HN_{Ser323}-OH_2-$O3_{PC1}$	31	$HZ1_{Lys376}$-$N1_{PC1}$	20
	HH_{Tyr354}-$O5_{PC1}$	18	$HZ2_{Lys376}$-$N1_{PC1}$	21
	$HD21_{Asn212}$-$O6_{PC1}$	18	$HZ3_{Lys376}$-$N1_{PC1}$	20
	$HD21_{Asn212}$-OH_2-$O6_{PC1}$	35	$OG1_{Thr365}$-H_{PC1}	39
	$HD22_{Asn212}$-OH_2-$O6_{PC1}$	41	O_{Thr350}-OH_2-$H1_{PC1}$	53
	HN_{Ser323}-OH_2-$O6_{PC1}$	33	O_{Val352}-OH_2-$H1_{PC1}$	49
	$OG1_{Ser323}$-H_2O-$O6_{PC1}$	51	OG_{Ser322}-$H2_{PC1}$	17
	$NE2_{His370}$-OH_2-$O7_{PC1}$	42	O_{Cys364}-$H4_{PC1}$	44

[a]Occupancies indicated are averages of the values obtained from the PC1 simulations
[b]Residues highlighted include Asn212, Ser322, Ser323, Gly324, Thr350, Val352, Tyr354, Cys364, Thr365, Ala368, Tyr369, His370 and Lys376

Table S5. Hydrophobic contacts observed for the PC1 simulations

Simulation	Hydrophobic Contact	Occupancy (%)	Hydrophobic Contact	Occupancy (%)
PC1[a,b]	CB_{Ala190}-C_{PC1}	28	CB_{Ala368}-$C10_{PC1}$	32
	CB_{Ala190}-$C1_{PC1}$	40	CB_{Ala368}-$C10_{PC1}$	58
	CB_{Ala190}-$C2_{PC1}$	53	CB_{Ala368}-$C12_{PC1}$	44
	CB_{Ala190}-$C3_{PC1}$	17	CB_{Tyr369}-$C12_{PC1}$	11
	$CD2_{Leu372}$-$C3_{PC1}$	40	CB_{Ala368}-$C13_{PC1}$	41
	$CD1_{Leu372}$-$C5_{PC1}$	68	CB_{Tyr369}-$C13_{PC1}$	12
	$CD2_{Leu372}$-$C5_{PC1}$	56	CB_{Ala368}-$C14_{PC1}$	52
	$CD2_{Leu372}$-$C6_{PC1}$	18	CB_{Tyr369}-$C14_{PC1}$	32
	CZ_{Phe211}-$C7_{PC1}$	23	CB_{Ala368}-$C15_{PC1}$	35
	$CE2_{Phe211}$-$C7_{PC1}$	23	CB_{Tyr369}-$C15_{PC1}$	64
	CB_{Ser189}-$C8_{PC1}$	11	$CD2_{His370}$-$C15_{PC1}$	13
	$CE2_{Phe211}$-$C8_{PC1}$	12	CB_{Tyr369}-$C16_{PC1}$	14
	$CE1_{Tyr354}$-$C8_{PC1}$	42	CB_{Tyr369}-$C17_{PC1}$	11

[a]Occupancies indicated are averages of the values obtained from the PC1 simulations
[b]Residues highlighted include Ser189, Ala190, Phe211, Tyr354, Ala368, Tyr369, His370 and Leu372.

Non-Bonded Interactions of Phosphodiester-Linked Inhibitor *(S)*-2

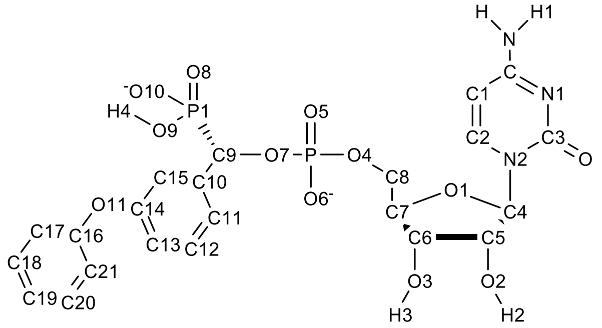

Table S6. Hydrogen bonding and water bridge interactions observed for the PC2 simulations

Simulation	Hydrogen Bonds	Occupancy (%)	Hydrogen Bonds	Occupancy (%)
PC2[a,b]	$HZ1_{Lys376}$-O_{PC2}	28	$HD22_{Asn233}$-$O10_{PC2}$	19
	$HZ2_{Lys376}$-O_{PC2}	28	$HD21_{Asn233}$-OH_2-$O10_{PC2}$	37
	$HZ3_{Lys376}$-O_{PC2}	29	$HE22_{Gln235}$-OH_2-$O10_{PC2}$	44
	NH_{Ser189}-$O1_{PC2}$	35	$HZ1_{Lys376}$-$N1_{PC2}$	21
	NH_{Gly324}-$O2_{PC2}$	35	$HZ2_{Lys376}$-$N1_{PC2}$	22
	$HD21_{Asn212}$-$O4_{PC2}$	34	$HZ3_{Lys376}$-$N1_{PC2}$	22
	$HD22_{Asn212}$-OH_2-$O4_{PC2}$	38	O_{Cys353}-H_{PC2}	26
	$HD21_{Asn212}$-$O5_{PC2}$	43	HN_{Gly191}-OH_2-H_{PC2}	32
	HN_{Ser323}-$O5_{PC2}$	12	O_{Thr350}-H_2O-H_{PC2}	31
	$HG1_{Ser323}$-$O5_{PC2}$	18	O_{Val352}-H_2O-H_{PC2}	52
	$HD22_{Asn212}$-$O8_{PC2}$	64	OG_{Thr365}-$H1_{PC2}$	24
	OH_{Tyr356}-HOH-$O8_{PC2}$	54	OG_{Ser322}-$H2_{PC2}$	20
	HH_{Tyr356}-OH_2-$O8_{PC2}$	33	O_{Tyr354}-$H4_{PC2}$	33
	$HD22_{Asn212}$-$O10_{PC2}$	12	-	-

[a]Occupancies indicated are averages of the values obtained from the PC2 simulations
[b]Residues highlighted include Ser189, Gly191, Asn212, Asn233, Gln235, Ser322, Ser323, Gly324, Thr350, Val352, Cys353, Tyr354, Tyr356, Thr365 and Lys376

Table S7. Hydrophobic contacts observed for the PC2 simulations

Simulation	Hydrophobic Contact	Occupancy (%)	Hydrophobic Contact	Occupancy (%)
PC2[a]	CB_{Ala190}-C_{PC2}	46[b]	$CE2_{Tyr354}$-$C13_{PC2}$	41[c]
	CB_{Ala190}-$C1_{PC2}$	62[b]	CZ_{Tyr354}-$C14_{PC2}$	12[c]
	$CD1_{Tyr354}$-$C1_{PC2}$	16[b]	$CE2_{Tyr354}$-$C14_{PC2}$	21[c]
	$CE1_{Tyr354}$-$C1_{PC2}$	22[b]	CZ_{Tyr354}-$C15_{PC2}$	25[c]
	CB_{Ala190}-$C2_{PC2}$	16[b]	$CE2_{Tyr354}$-$C15_{PC2}$	11[c]
	$CE1_{Tyr354}$-$C2_{PC2}$	24[b]	CG_{Asn233}-$C17_{PC2}$	17[d]
	$CD2_{Leu372}$-$C3_{PC2}$	22[b]	CB_{Gln235}-$C17_{PC2}$	11[d]
	$CD1_{Leu372}$-$C5_{PC2}$	21[b]	$CE1_{Tyr275}$-$C17_{PC2}$	30[d]
	$CD2_{Leu372}$-$C5_{PC2}$	49[b]	CZ_{Tyr275}-$C17_{PC2}$	23[d]
	$CD2_{Leu372}$-$C6_{PC2}$	21[b]	CB_{Asn233}-$C18_{PC2}$	55[d]
	CZ_{Phe211}-$C7_{PC2}$	30[b]	CG_{Asn233}-$C18_{PC2}$	25[d]
	$CE2_{Phe211}$-$C7_{PC2}$	14[b]	CB_{Gln235}-$C18_{PC2}$	26[d]
	$CE2_{Phe211}$-$C8_{PC2}$	37[b]	CD_{Pro259}-$C18_{PC2}$	28[d]
	$CE1_{Tyr354}$-$C8_{PC2}$	23[b]	CG_{Pro259}-$C18_{PC2}$	33[d]
	$CE1_{Tyr354}$-$C9_{PC2}$	18[c]	$CE1_{Tyr275}$-$C18_{PC2}$	36[d]
	$CE1_{Tyr354}$-$C10_{PC2}$	23[c]	CB_{Asn233}-$C19_{PC2}$	16[d]
	CZ_{Tyr354}-$C10_{PC2}$	55[c]	CG_{Asn233}-$C19_{PC2}$	21[d]
	$CE2_{Tyr354}$-$C10_{PC2}$	14[c]	$CZ2_{Trp257}$-$C19_{PC2}$	54[d]
	CG_{Tyr354}-$C11_{PC2}$	13[c]	$CH2_{Trp257}$-$C19_{PC2}$	52[d]
	$CD1_{Tyr354}$-$C11_{PC2}$	19[c]	CD_{Pro259}-$C19_{PC2}$	66[d]
	$CE1_{Tyr354}$-$C11_{PC2}$	41[c]	CG_{Pro259}-$C19_{PC2}$	42[d]
	$CD2_{Tyr354}$-$C11_{PC2}$	33[c]	CB_{Asn233}-$C20_{PC2}$	48[d]
	$CE2_{Tyr354}$-$C11_{PC2}$	65[c]	CG_{Asn233}-$C20_{PC2}$	11[d]
	CG_{Tyr354}-$C12_{PC2}$	14[c]	$CH2_{Trp257}$-$C20_{PC2}$	58[d]
	CZ_{Tyr354}-$C12_{PC2}$	36[c]	$CE1_{Tyr369}$-$C20_{PC2}$	51[d]
	$CD2_{Tyr354}$-$C12_{PC2}$	46[c]	$CD1_{Tyr369}$-$C21_{PC2}$	28[d]
	$CE2_{Tyr354}$-$C12_{PC2}$	66[c]	$CE1_{Tyr369}$-$C21_{PC2}$	51[d]
	$CD2_{Tyr354}$-$C13_{PC2}$	16[c]	$CE1_{His370}$-$C21_{PC2}$	31[d]

[a]Residues highlighted include Ala190, Phe211, Asn233, Gln235, Trp257, Pro259, Tyr275, Tyr354, Tyr369, His370 and Leu372.
[b]Occupancies indicated are averages of the values obtained from all of the PC2 simulations.
[c]Occupancies indicated are the values obtained from the PC2-2000 simulation.
[d]Occupancies indicated are averages of the values obtained from the PC2-2500 and PC2-3000 simulations.

NOTE: All of the hydrophobic contacts of the cytidine moiety were consistent across all three PC2 simulations.

Non-Bonded Interactions of Triazole-Linked Inhibitor *(R)*-3

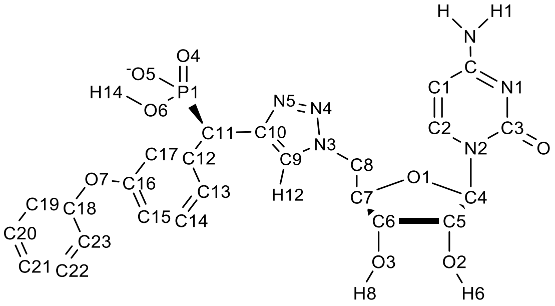

Table S8. Hydrogen bonding and water bridge interactions observed for the TC1 simulations

Simulation	Hydrogen Bonds	Occupancy (%)	Hydrogen Bonds	Occupancy (%)
TC1[a,b]	$HZ1_{Lys376}$-O_{TC1}	28	$HD22_{Asn212}$-$N5_{TC1}$	10
	$HZ2_{Lys376}$-O_{TC1}	27	$OG1_{Thr365}$-H_{TC1}	15
	$HZ3_{Lys376}$-O_{TC1}	28	$OG1_{Thr365}$-H_2O- H_{TC1}	33
	HN_{Gly324}-$O2_{TC1}$	29	HN_{Gly191}-H_2O-$H1_{TC1}$	43
	HH_{Tyr354}-$O4_{TC1}$	22	O_{Thr350}-H_2O-$H1_{TC1}$	51
	$HZ1_{Lys376}$-$N1_{TC1}$	24	O_{Val352}-H_2O-$H1_{TC1}$	41
	$HZ2_{Lys376}$-$N1_{TC1}$	23	O_{Cys353}-$H1_{TC1}$	16
	$HZ3_{Lys376}$-$N1_{TC1}$	23	OG_{Ser322}-$H6_{TC1}$	35
	$HD21_{Asn212}$-$N4_{TC1}$	20	OG_{Ser322}-$H8_{TC1}$	18
	$HD22_{Asn212}$-$N4_{TC1}$	18	OH_{Tyr354}-$H12_{TC1}$	26
	$HD21_{Asn212}$-$N5_{TC1}$	17	$NE2_{His370}$-$H14_{TC1}$	30

[a]Occupancies indicated are averages of the values obtained from the TC1 simulations
[b]Residues highlighted include Gly191, Asn212, Ser322, Gly324, Thr350, Val352, Cys353, Tyr354, Thr365, His370 and Lys376

Table S9. Hydrophobic contacts observed for the TC1 simulations

Simulation	Hydrophobic Contact	Occupancy (%)	Hydrophobic Contact	Occupancy (%)
TC1[a,b]	CB_{Ala190}-C_{TC1}	66	CG_{Tyr369}-$C15_{TC1}$	26
	CB_{Ala190}-$C1_{TC1}$	29	$CD1_{Tyr369}$-$C15_{TC1}$	28
	$CD1_{Tyr354}$-$C1_{TC1}$	11	$CE1_{Tyr369}$-$C18_{TC1}$	13
	$CE1_{Tyr354}$-$C1_{TC1}$	14	CB_{Gln235}-$C19_{TC1}$	17
	CB_{Ala190}-$C2_{TC1}$	17	CB_{Asn233}-$C20_{TC1}$	24
	$CE1_{Tyr354}$-$C2_{TC1}$	13	CB_{Gln235}-$C20_{TC1}$	18
	$CD2_{Leu372}$-$C3_{TC1}$	15	$CZ2_{Trp257}$-$C20_{TC1}$	16
	CZ_{Phe211}-$C4_{TC1}$	36	$CH2_{Trp257}$-$C20_{TC1}$	13
	$CE2_{Phe211}$-$C4_{TC1}$	25	CD_{Pro259}-$C20_{TC1}$	10
	$CD1_{Leu372}$-$C5_{TC1}$	59	CG_{Pro259}-$C20_{TC1}$	18
	$CD2_{Leu372}$-$C5_{TC1}$	44	$CE1_{Tyr275}$-$C20_{TC1}$	11
	$CD2_{Leu372}$-$C6_{TC1}$	35	CB_{Asn233}-$C21_{TC1}$	15
	CZ_{Phe211}-$C7_{TC1}$	23	CD_{Pro259}-$C21_{TC1}$	38
	$CE2_{Phe211}$-$C7_{TC1}$	48	CG_{Pro259}-$C21_{TC1}$	37
	$CE2_{Phe211}$-$C8_{TC1}$	22	$CE1_{Tyr275}$-$C21_{TC1}$	11
	$CE1_{Tyr354}$-$C8_{TC1}$	14	CB_{Asn233}-$C22_{TC1}$	19
	CZ_{Tyr354}-$C9_{TC1}$	12	CB_{Gln235}-$C22_{TC1}$	22
	$CE1_{Tyr369}$-$C12_{TC1}$	15	$CZ2_{Trp257}$-$C22_{TC1}$	13
	$CE1_{His370}$-$C12_{TC1}$	12	$CH2_{Trp257}$-$C22_{TC1}$	14
	$CD2_{His370}$-$C13_{TC1}$	20	CG_{Pro259}-$C22_{TC1}$	14
	CB_{Tyr369}-$C14_{TC1}$	18	$CE1_{Tyr275}$-$C22_{TC1}$	23
	$CD1_{Tyr369}$-$C14_{TC1}$	21	CZ_{Tyr275}-$C22_{TC1}$	11
	$CD2_{His370}$-$C14_{TC1}$	39	$CE2_{Tyr369}$-$C22_{TC1}$	13
	CG_{Gln235}-$C15_{TC1}$	17	CB_{Gln235}-$C23_{TC1}$	19
	CB_{Tyr369}-$C15_{TC1}$	24	$CE1_{Tyr369}$-$C23_{TC1}$	16

[a]Occupancies indicated are averages of the values obtained from the TC1 simulations
[b]Residues highlighted include Ala190, Phe211, Asn233, Gln235, Trp257, Pro259, Tyr275, Tyr354, Tyr369, His370 and Leu372

Non-Bonded Interactions of Triazole-Linked Inhibitor *(S)*-3

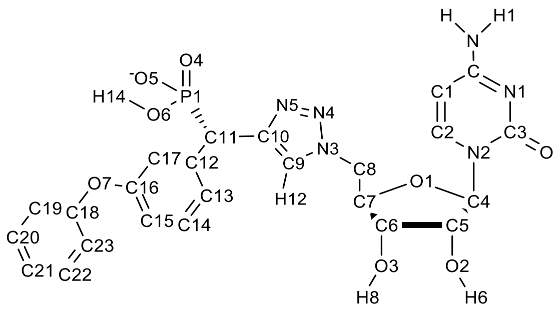

Table S10. Hydrogen bonding and water bridge interactions observed for the TC2 simulations

Simulation	Hydrogen Bonds	Occupancy (%)	Hydrogen Bonds	Occupancy (%)
TC2[a,b]	$HZ1_{Lys376}$-O_{TC2}	30	$HZ3_{Lys376}$-$N1_{TC2}$	22
	$HZ2_{Lys376}$-O_{TC2}	30	$HD22_{Asn212}$-$N4_{TC2}$	10
	$HZ3_{Lys376}$-O_{TC2}	26	$NE2_{His370}$-H_2O-$N4_{TC2}$	43
	HN_{Gly324}-$O2_{TC2}$	43	$HD21_{Asn212}$-$N5_{TC2}$	28
	HH_{Tyr354}-$O4_{TC2}$	21	HN_{Ser323}-H_2O-$N5_{TC2}$	35
	HH_{Tyr354}-OH_2-$O4_{TC2}$	33	$HG1_{Ser323}$-H_2O-$N5_{TC2}$	34
	HH_{Tyr354}-$O5_{TC2}$	14	O_{Cys353}-H_{TC2}	14
	HN_{Ala368}-OH_2-$O5_{TC2}$	30	HN_{Gly191}-H_2O-H_{TC2}	45
	HN_{His370}-OH_2-$O5_{TC2}$	30	$OG1_{Thr365}$-$H1_{TC2}$	15
	$HZ1_{Lys376}$-$N1_{TC2}$	26	OG_{Ser322}-$H6_{TC2}$	31
	$HZ2_{Lys376}$-$N1_{TC2}$	27	OH_{Tyr354}-$H12_{TC2}$	22

[a]Occupancies indicated are averages of the values obtained from the TC2 simulations
[b]Residues highlighted include Gly191, Asn212, Ser322, Ser323, Gly324, Cys353, Tyr354, Thr365, Ala368, His370 and Lys376

Table S11. Hydrophobic contacts observed for the TC2 simulations

Simulation	Hydrophobic Contact	Occupancy (%)	Hydrophobic Contact	Occupancy (%)
TC2[a,b]	CB_{Ala190}-C_{TC2}	44	CB_{Asn233}-$C20_{TC2}$	12
	CB_{Ala190}-$C1_{TC2}$	53	CB_{Gln235}-$C20_{TC2}$	12
	$CD1_{Tyr354}$-$C1_{TC2}$	12	CG_{Pro259}-$C20_{TC2}$	18
	$CE1_{Tyr354}$-$C1_{TC2}$	18	$CE1_{Tyr275}$-$C20_{TC2}$	11
	CB_{Cys364}-$C1_{TC2}$	33	$CE1_{Tyr369}$-$C20_{TC2}$	18
	CB_{Ala190}-$C2_{TC2}$	31	CEZ_{Tyr369}-$C20_{TC2}$	15
	$CE1_{Tyr354}$-$C2_{TC2}$	20	$CE1_{His370}$-$C20_{TC2}$	15
	CB_{Cys364}-$C2_{TC2}$	14	CB_{Asn233}-$C21_{TC2}$	11
	$CD2_{Leu372}$-$C3_{TC2}$	10	CB_{Gln235}-$C21_{TC2}$	10
	CZ_{Phe211}-$C4_{TC2}$	34	$CH2_{Trp257}$-$C21_{TC2}$	14
	$CE2_{Phe211}$-$C4_{TC2}$	23	CG_{Pro259}-$C21_{TC2}$	45
	$CD1_{Leu372}$-$C5_{TC2}$	54	$CE1_{Tyr275}$-$C21_{TC2}$	10
	$CD2_{Leu372}$-$C5_{TC2}$	43	$CE1_{Tyr369}$-$C21_{TC2}$	39
	$CD2_{Leu372}$-$C6_{TC2}$	25	CEZ_{Tyr369}-$C21_{TC2}$	26
	CZ_{Phe211}-$C7_{TC2}$	22	CB_{Asn233}-$C22_{TC2}$	12
	$CE2_{Phe211}$-$C7_{TC2}$	28	CB_{Gln235}-$C22_{TC2}$	15
	$CE2_{Phe211}$-$C8_{TC2}$	18	$CZ2_{Trp257}$-$C22_{TC2}$	13
	$CE1_{Tyr354}$-$C8_{TC2}$	33	$CH2_{Trp257}$-$C22_{TC2}$	13
	CZ_{Tyr354}-$C8_{TC2}$	10	CG_{Pro259}-$C22_{TC2}$	23
	CZ_{Tyr354}-$C9_{TC2}$	17	$CE1_{Tyr275}$-$C22_{TC2}$	13
	$CD2_{His370}$-$C11_{TC2}$	18	$CE1_{Tyr369}$-$C22_{TC2}$	17
	CB_{Leu236}-$C14_{TC2}$	12	CZ_{Tyr369}-$C22_{TC2}$	12
	CB_{Gln235}-$C18_{TC2}$	17	$CE1_{His370}$-$C22_{TC2}$	20
	$CE1_{Tyr369}$-$C18_{TC2}$	11	CB_{Asn233}-$C23_{TC2}$	13
	CB_{Gln235}-$C19_{TC2}$	22	CB_{Gln235}-$C23_{TC2}$	22
	$CE1_{Tyr369}$-$C19_{TC2}$	10	-	-

[a]Occupancies indicated are averages of the values obtained from the TC2 simulations
[b]Residues highlighted include Ala190, Phe211, Asn233, Leu236, Gln235, Trp257, Pro259, Tyr275, Tyr354, Cys364, Tyr369, His370 and Leu372

Non-Bonded Interactions of Carbamate-Linked Inhibitor *(R)*-4

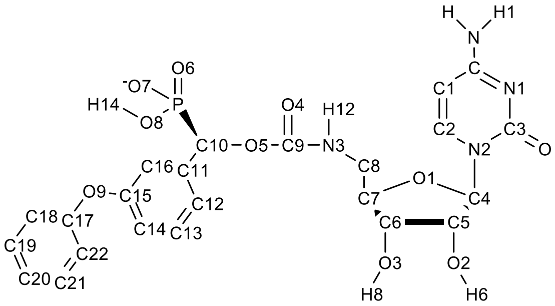

Table S12. Hydrogen bonding and water bridge interactions observed for the CC1 simulations

Simulation	Hydrogen Bonds	Occupancy (%)	Hydrogen Bonds	Occupancy (%)
CC1[a,b]	$HZ1_{Lys376}$-O_{CC1}	20	HN_{Tyr369}-$O7_{CC1}$	43
	$HZ2_{Lys376}$-O_{CC1}	24	HN_{His370}-$O7_{CC1}$	43
	$HZ3_{Lys376}$-O_{CC1}	19	HN_{Ala368}-$O8_{CC1}$	34
	$HG1_{Ser189}$-$O2_{CC1}$	10	$HZ1_{Lys376}$-$N1_{CC1}$	20
	HH_{Tyr354}-$O4_{CC1}$	29	$HZ2_{Lys376}$-$N1_{CC1}$	23
	HN_{Ala368}-$O6_{CC1}$	11	$HZ3_{Lys376}$-$N1_{CC1}$	19
	HN_{Tyr369}-$O6_{CC1}$	53	O_{Val352}-H_2O-H_{CAR}	31
	HN_{His370}-$O6_{CC1}$	35	$OG1_{Thr365}$-$H1_{CC1}$	18
	HN_{Ala368}-$O7_{CC1}$	19	OG_{Ser188}-$H6_{CC1}$	34

[a]Occupancies indicated are averages of the values obtained from the CC1 simulations
[b]Residues highlighted include Ser188, Ser189, Val 352, Tyr354, Thr365, Ala368, Tyr369, His370 and Lys376

Table S13. Hydrophobic contacts observed for the CC1 simulations

Simulation	Hydrophobic Contact	Occupancy (%)	Hydrophobic Contact	Occupancy (%)
CC1[a,b]	CB_{Ala190}-C_{CC1}	16	$CD2_{His370}$-$C9_{CC1}$	14
	CB_{Ala190}-$C1_{CC1}$	28	$CD2_{His370}$-$C16_{CC1}$	53
	CB_{Cys364}-$C1_{CC1}$	27	$CD1_{Tyr369}$-$C17_{CC1}$	33
	CB_{Ala190}-$C2_{CC1}$	28	$CE1_{Tyr369}$-$C17_{CC1}$	25
	CB_{Ala190}-$C3_{CC1}$	28	$CE1_{His370}$-$C17_{CC1}$	11
	CB_{Ser322}-$C6_{CC1}$	27	CG_{Tyr369}-$C18_{CC1}$	16
	$CD2_{Leu372}$-$C6_{CC1}$	18	$CD1_{Tyr369}$-$C18_{CC1}$	20
	CZ_{Phe211}-$C7_{CC1}$	15	$CE1_{Tyr369}$-$C18_{CC1}$	20
	$CE2_{Phe211}$-$C7_{CC1}$	23	CB_{Asn233}-$C20_{CC1}$	17
	$CE2_{Phe211}$-$C8_{CC1}$	21	CG_{Tyr369}-$C22_{CC1}$	12
	CB_{Ser322}-$C8_{CC1}$	15	$CD1_{Tyr369}$-$C22_{CC1}$	22
	$CD1_{Leu372}$-$C8_{CC1}$	18	$CE1_{Tyr369}$-$C22_{CC1}$	26
	$CD2_{Leu372}$-$C8_{CC1}$	12	-	-

[a]Occupancies indicated are averages of the values obtained from the CC1 simulations
[b]Residues highlighted include Ala190, Phe211, Asn233, Ser322, Cys364, Tyr369, His370 and Leu372

Non-Bonded Interactions of Carbamate-Linked Inhibitor *(S)*-4

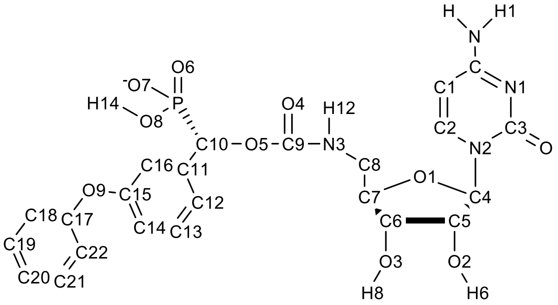

Table S14. Hydrogen bonding and water bridge interactions observed for the CC2 simulations

Simulation	Hydrogen Bonds	Occupancy (%)	Hydrogen Bonds	Occupancy (%)
CC2[a,b]	HG1$_{\text{Thr365}}$-O$_{\text{CC2}}$	15	HN$_{\text{Ser323}}$-OH$_2$-O7$_{\text{CC2}}$	32
	HZ1$_{\text{Lys376}}$-O$_{\text{CC2}}$	35	HG1$_{\text{Thr365}}$-N1$_{\text{CC2}}$	21
	HZ2$_{\text{Lys376}}$-O$_{\text{CC2}}$	31	HZ1$_{\text{Lys376}}$-N1$_{\text{CC2}}$	18
	HZ3$_{\text{Lys376}}$-O$_{\text{CC2}}$	26	HZ2$_{\text{Lys376}}$-N1$_{\text{CC2}}$	19
	NH$_{\text{Gly324}}$-O2$_{\text{CC2}}$	43	HZ3$_{\text{Lys376}}$-N1$_{\text{CC2}}$	16
	NH$_{\text{Ser323}}$-O3$_{\text{CC2}}$	22	O$_{\text{Cys353}}$-H$_{\text{CC2}}$	26
	NH$_{\text{Gly324}}$-O3$_{\text{CC2}}$	32	OG1$_{\text{Thr365}}$-H1$_{\text{CC2}}$	24
	HD21$_{\text{Asn212}}$-O4$_{\text{CC2}}$	12	O$_{\text{Thr365}}$-H1$_{\text{CC2}}$	11
	HD22$_{\text{Asn212}}$-O4$_{\text{CC2}}$	43	OG$_{\text{Ser322}}$-H6$_{\text{CC2}}$	25
	HH$_{\text{Tyr354}}$-O4$_{\text{CC2}}$	15	OG1$_{\text{Ser189}}$-H12$_{\text{CC2}}$	18
	HH$_{\text{Tyr354}}$-O6$_{\text{CC2}}$	11	-	-

[a]Occupancies indicated are averages of the values obtained from the CC2 simulations
[b]Residues highlighted include Ser189, Asn212, Ser322, Ser323, Gly324, Cys353, Tyr354, Thr365 and Lys376

Table S15. Hydrophobic contacts observed for the CC2 simulations

Simulation	Hydrophobic Contact	Occupancy (%)	Hydrophobic Contact	Occupancy (%)
CC2[a,b]	CB_{Ala190}-C_{CC2}	54	$CD2_{Phe211}$-$C8_{CC2}$	41
	CB_{Ala190}-$C1_{CC2}$	34	CZ_{Tyr354}-$C11_{CC2}$	13
	$CD1_{Tyr354}$-$C1_{CC2}$	27	$CD2_{His370}$-$C12_{CC2}$	22
	$CE1_{Tyr354}$-$C1_{CC2}$	12	$CD2_{Leu236}$-$C13_{CC2}$	11
	$CE1_{Tyr354}$-$C2_{CC2}$	15	CB_{Cys353}-$C13_{CC2}$	20
	$CD2_{Leu372}$-$C3_{CC2}$	14	CB_{Cys364}-$C13_{CC2}$	10
	$CD1_{Leu372}$-$C5_{CC2}$	14	CB_{Cys364}-$C14_{CC2}$	11
	$CD2_{Leu372}$-$C5_{CC2}$	11	$CE1_{Tyr354}$-$C16_{CC2}$	31
	$CD2_{Phe211}$-$C7_{CC2}$	31	$CD2_{Tyr354}$-$C17_{CC2}$	17
	CB_{Ser189}-$C8_{CC2}$	25	$CE2_{Tyr354}$-$C18_{CC2}$	24
	CB_{Phe211}-$C8_{CC2}$	16	$CD2_{Tyr354}$-$C22_{CC2}$	10
	CG_{Phe211}-$C8_{CC2}$	39	$CE2_{Tyr354}$-$C22_{CC2}$	11

[a]Occupancies indicated are averages of the values obtained from the CC1 simulations
[b]Residues highlighted include Ser189, Ala190, Phe211, Leu236, Cys353, Tyr354, Cys364, His370 and Leu372

Cluster Analysis of Complexed MD Simulations

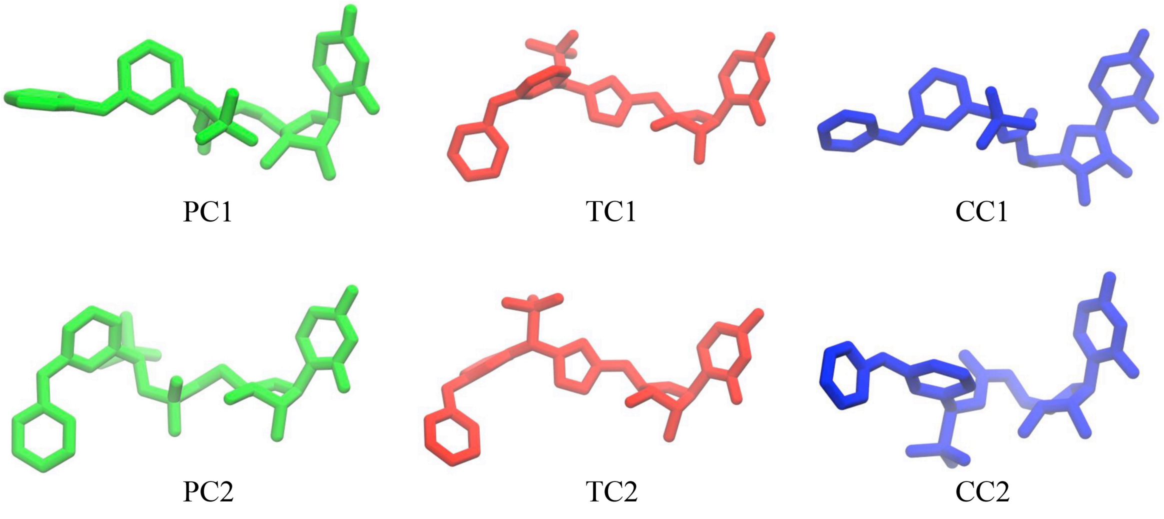

Figure S9. The most populated clusters of each respective ligand from the human ST6Gal I-ligand complex simulations. Clusters were generated using combined trajectories that represented every 10 ps of the triplicate simulations during their 90 ns production runs (i.e. 27,000 structures in total per simulation type). The clusters shown represent the following percentage of simulation time: PC1 68%; TC1 49%; CC1 31%; PC2 67%; TC2 42%; CC2 67%.

Cluster Analysis of Ligand MD Simulations

A

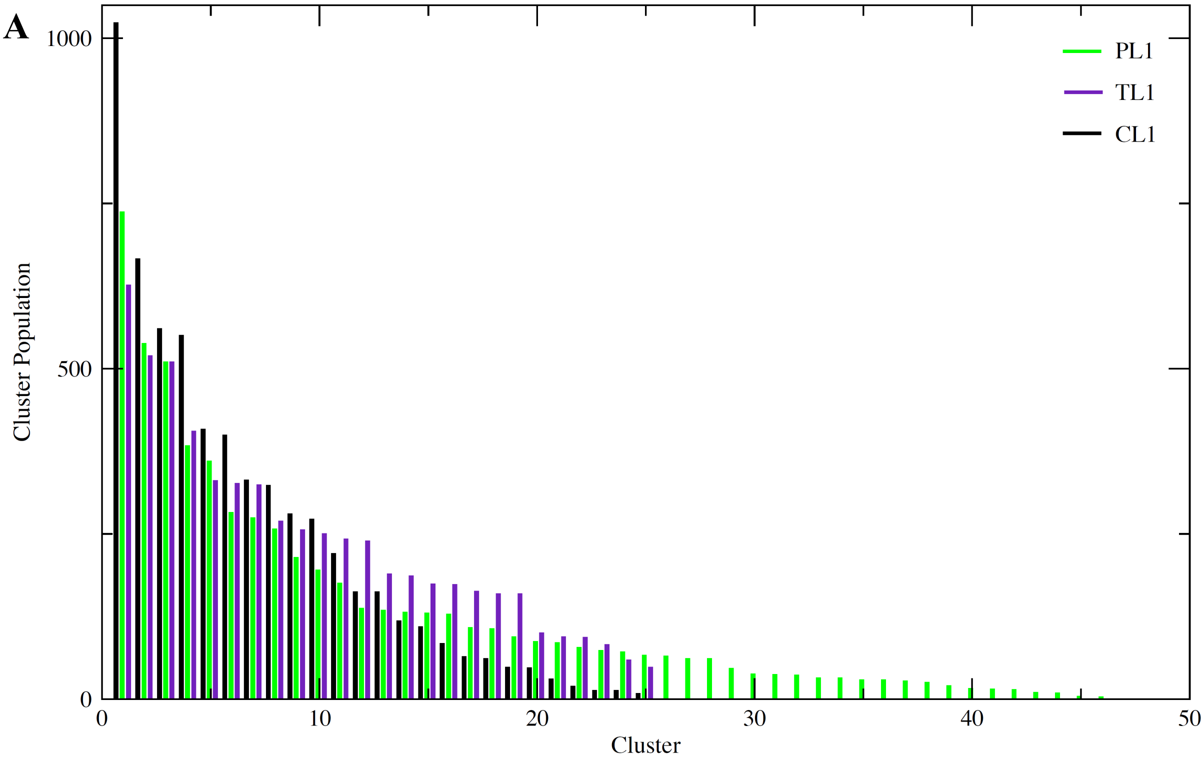

B

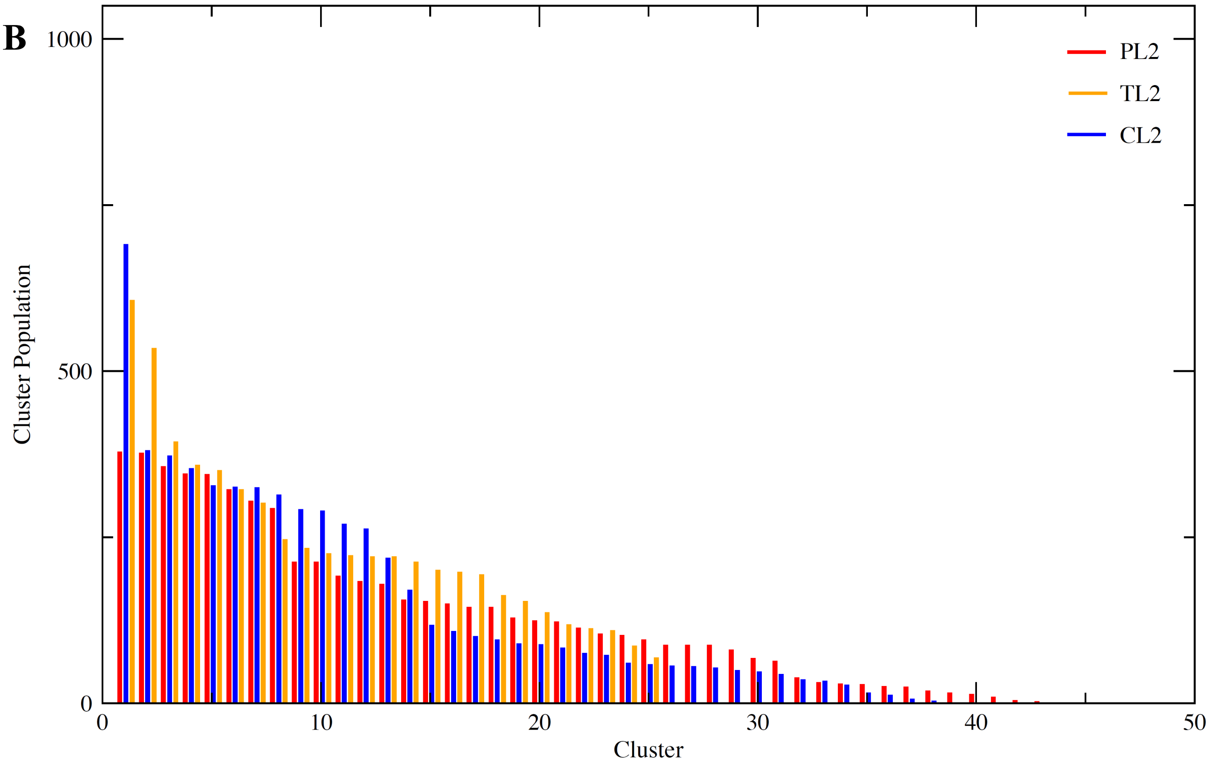

Figure S10. The distribution of cluster population for the *(R)*- **(A)** and *(S)*-inhibitors **(B)** in water box MD simulations. Clusters were generated using combined trajectories that represented every 10 ps of the triplicate simulations (i.e. 6,000 structures in total per simulation type).
